# The neuro-immuno-metabolic axis of exercise: a unified mechanistic framework for exercise-induced cognitive enhancement and psychological resilience

**DOI:** 10.3389/fpsyg.2026.1773561

**Published:** 2026-03-09

**Authors:** Yinghao Shen, Zhujun Mao, Yupeng Yang, Heming Chen, Hongda Wang, Xi Cheng, Wenyue Zhu, Junjie Liu, Chunxiao Wang

**Affiliations:** 1 Graduate School, Harbin Sport University, Harbin, China; 2 School of Basic Medicine, China Three Gorges University, Yichang, China; 3 College of Science and Technology, China Three Gorges University, Yichang, China; 4 School of Exercise Science and Health, Harbin Sport University, Harbin, China

**Keywords:** cognitive enhancement, exercise, exercise modality specificity, gut-brain axis, neuro-immuno-metabolic axis, precision exercise medicine, psychological resilience, repair-oriented inflammation

## Abstract

**Background:**

Physical exercise is widely recognized for promoting cognitive function and psychological resilience; however, the precise systemic mechanisms remain fragmented across isolated disciplines. Existing models often fail to capture the complex, multi-systemic nature of these adaptations.

**Methods:**

We conducted a comprehensive literature search in PubMed, Web of Science, and Scopus databases up to December 2025. Keywords included “exercise,” “neuroinflammation,” “metabolism,” “kynurenine pathway,” and “gut-brain axis.” We prioritized high-quality preclinical and clinical studies that examined bidirectional cross-talk between at least two physiological systems (neural, immune, or metabolic) to construct a unified theoretical synthesis.

**Results:**

Based on this synthesis, we propose the “Neuro-Immuno-Metabolic (NIM) Axis.” Unlike linear bipartite models, this framework positions exercise as a systemic “energy challenge” that triggers a coordinated recalibration. Key mechanisms identified include: (1) Metabolic Signaling: Lactate, ketone bodies, and the PGC-1α-mediated kynurenine detoxification pathway act as systemic signalers; (2) Immune Regulation: Exercise drives a shift from pro-inflammatory surveillance to “repair-oriented inflammation” rather than binary M1/M2 phenotypes; and (3) Gut-Brain Integration: Gut metabolites (e.g., indoles, SCFAs) regulate central immune tolerance via AhR signaling. Furthermore, we delineate the mechanistic specificity of aerobic, resistance, and high-intensity interval training (HIIT).

**Conclusion:**

The NIM axis provides a novel, integrative framework that explains how metabolic stress is transduced into psychological resilience. These insights establish a theoretical foundation for precision exercise medicine and advocate for future multi-omics research to develop individualized interventions.

## Graphical abstract

The neuro-immuno-metabolic (NIM) axis framework. Schematic representation of the Neuro-Immuno-Metabolic (NIM) Axis, illustrating how specific exercise modalities (Resistance, Aerobic, HIIT) act as systemic energy triggers to modulate metabolic signaling, gut-brain integration, and immune regulation, ultimately promoting cognitive function and psychological resilience. 
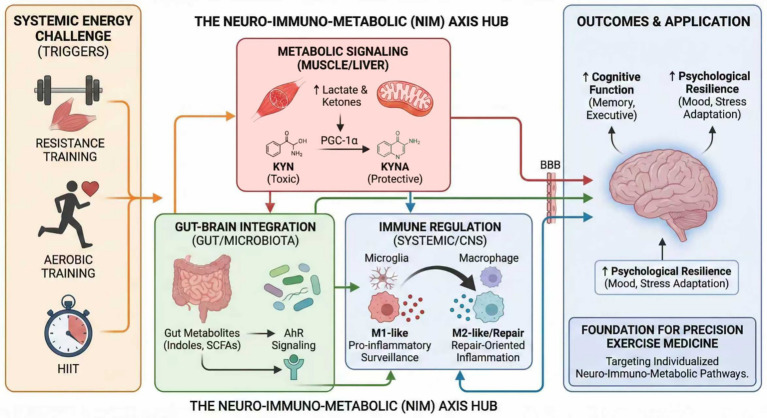

## 1 Introduction

The positive impact of physical exercise on cognitive function and mental health has been extensively documented across diverse populations, age groups, and clinical conditions. It underscores exercise’s significance as a non-pharmacological intervention to enhance brain health and psychological well-being. Clinical and observational studies confirm that exercise improves specific cognitive domains including memory, executive function, attention, and cognitive flexibility. Benefits are observed in healthy individuals, older adults, adolescents, and patients with neurodegenerative or psychiatric disorders (Cohn-Schwartz, 2020; Smith and Merwin, 2021; Xu et al., 2024). For instance, narrative reviews highlight that physical activity enhances cerebral blood flow. It stimulates neurotrophic factor synthesis and reduces oxidative stress and inflammation. These effects collectively contribute to improved memory and executive functioning in adults and academic performance in younger populations (Biazus-Sehn et al., 2020; Pujari, 2024). These neurobiological effects are accompanied by mood-regulating benefits. Activation of the endocannabinoid system and release of endorphins mediate these benefits, alleviating symptoms of anxiety and depression. In addition, exercise has been investigated as an adjunctive therapy in mental illnesses such as major depressive disorder (MDD), generalized anxiety disorder, schizophrenia, bipolar disorder, and eating disorders. It improves treatment efficacy and overall quality of life (Herbert et al., 2020; Pujari, 2024).

Multimodal brain imaging advances have further elucidated structural and functional brain adaptations. These changes underpin exercise-induced improvements in cognition and mental health (Valk et al., 2022). Cross-sectional studies using multimodal imaging techniques yield key findings. Individuals with regular exercise habits show greater gray matter volume in frontal and hippocampal regions. They also exhibit enhanced white matter integrity and more robust functional network configurations in key brain networks such as the default mode network (Yao et al., 2024). Notably, these neural correlates are associated with lower levels of depression and anxiety. They also link to higher life satisfaction, emphasizing the role of an active lifestyle in fostering psychological resilience and cognitive robustness. Such findings contribute to a growing body of evidence supporting exercise as a vital component in mental health promotion and cognitive maintenance (Gray et al., 2020; Zhao et al., 2020).

In clinical populations, structured exercise interventions have demonstrated efficacy in ameliorating physical and mental health outcomes. For example, randomized controlled trials implemented balance and muscle-strengthening programs during the COVID-19 pandemic. These included the Otago Exercise Programme for elderly individuals with cognitive frailty. The trials showed significant improvements in physical function, reduced depressive symptoms, and enhanced mental health-related quality of life (He et al., 2025; Kamiya et al., 2025; Tuan et al., 2024). Furthermore, among adolescents, both chronic participation in sports and acute bouts of exercise yield benefits in mental health and executive functioning. Differential effects are observed based on sport type and exercise intensity (Pasquerella et al., 2025; Song et al., 2024). These age- and condition-specific findings underscore the importance of tailoring exercise interventions to optimize cognitive and psychological outcomes.

Extensive reviews confirm physical activity’s protective effects against age-related cognitive decline. These studies highlight benefits of aerobic, resistance, and combined training modalities in older adults (Dos Santos et al., 2020; Zare et al., 2025). Moderate-intensity aerobic exercise (typically defined as 64–76% of maximum heart rate) enhances memory, executive function, and mood regulation. It potentially acts via increased hippocampal neurogenesis and elevated brain-derived neurotrophic factor (BDNF) levels (Antunes et al., 2020; Enette et al., 2020). Resistance training acutely improves visuospatial processing and executive functions. Chronic resistance regimens promote neurogenesis and enhance cerebral blood flow to prefrontal regions. Importantly, findings related to concurrent training are heterogeneous. Emerging evidence suggests that combining concurrent exercise with cognitive tasks yields superior cognitive and mental health benefits compared to aerobic exercise alone (Balbim et al., 2024; Dhahbi et al., 2025; Kraemer and Kraemer, 2023). These insights advocate for incorporating diverse exercise modalities in geriatric cognitive health strategies.

Meta-analyses and umbrella reviews further consolidate the evidence base. They demonstrate that exercise interventions produce small to moderate cognitive improvements across age groups and health statuses. Notably, in children and adolescents with attention-deficit/hyperactivity disorder (ADHD), exercise enhances cognitive flexibility, inhibitory control, and inattention symptoms. Effects on emotional and social functioning remain less robust (Dastamooz et al., 2023). Exercise’s impact on cognitive domains is influenced by multiple factors. These include exercise type, intensity, duration, and frequency. Individual characteristics such as sex and genetic polymorphisms (e.g., the BDNF Val66Met variant) are also key (Liu et al., 2020; Ludyga et al., 2020). These findings call for further research to delineate optimal exercise prescriptions and mechanisms underlying individual variability in response.

Central to the neurobiological mechanisms of exercise-induced cognitive enhancement is the role of BDNF. This neurotrophin is critical for synaptic plasticity, neurogenesis, and neuronal survival. Exercise elevates peripheral and central BDNF levels. This elevation correlates with improved cognitive function in healthy individuals. It also benefits patients with cognitive impairments such as mild cognitive impairment and Alzheimer’s disease (Huang et al., 2021; Oyovwi et al., 2025). Experimental studies reveal that exercise modulates epigenetic markers such as H3K9me3 at BDNF promoter regions. This regulation of BDNF expression occurs in an age-dependent manner (Ionescu-Tucker et al., 2021). Additionally, BDNF mediates exercise-induced shifts in amyloid precursor protein processing. It reduces Alzheimer’s disease pathology and enhances recognition memory (Baranowski et al., 2023). These molecular adaptations highlight BDNF as a key integrator of exercise effects on brain plasticity and cognitive resilience.

Beyond BDNF, exercise exerts multifaceted effects through neuroimmune and metabolic pathways. It challenges the traditional view of neuroinflammation as solely detrimental. The concept of “repair-oriented” or “resolving” inflammation has emerged. Exercise modulates immune responses through this concept to promote neuroprotection and tissue repair. For instance, exercise increases regulatory T cell populations. These populations facilitate white matter repair post-stroke (Mu et al., 2025). Exercise also modulates microglial lipid metabolism via the AMPK-PGC1α-PPARγ pathway. This modulation attenuates neuroinflammation after spinal cord injury (Ying et al., 2025). Exercise further orchestrates systemic metabolic and neuroimmune homeostasis via the brain-muscle-liver axis. It enhances mitochondrial function, reduces oxidative stress, and balances pro- and anti-inflammatory cytokines. These effects slow aging and neurodegeneration (Kong et al., 2025).

Recent advances have expanded understanding of the gut-brain-muscle axis. They reveal bidirectional communication among the central nervous system, skeletal muscle, and gut microbiota. Specifically, exercise influences gut microbial composition and metabolite production. These changes in turn modulate muscle metabolism, neuroinflammation, and cognitive function (Cutuli et al., 2023; Morella et al., 2023; Ribeiro et al., 2022). Myokines produced during muscle contraction mediate these effects. Key examples include irisin and BDNF. These myokines promote neuroplasticity and systemic health (Nicastri et al., 2022; Wang et al., 2022). This integrative perspective underscores the complexity of exercise’s neurobiological impact. It involves neuro-immune-metabolic crosstalk across multiple organ systems.

Importantly, the efficacy of exercise in enhancing cognition and mental health is influenced by exercise modality, intensity, duration, and individual factors. Network meta-analyses identify effective mind–body exercise modes. Pilates and Tai Chi benefit chronic non-specific low back pain and cognitive function (Shi et al., 2022). Similarly, high-intensity training improves outcomes in chronic nonspecific low back pain (Verbrugghe et al., 2020). Acute vigorous exercise elevates lactate and BDNF levels more than moderate exercise, leading to greater cognitive benefits (Waddington et al., 2024). Moreover, sex differences modulate exercise-induced cognitive and molecular responses, necessitating sex-specific exercise recommendations (Kotecki and Bradford, 2022; Short et al., 2022). These findings advocate for precision exercise medicine, tailoring interventions to optimize neurobiological and psychological outcomes.

In summary, the accumulated evidence reveals that physical exercise enhances cognitive function and mental health through a complex, integrated neuro-immune-metabolic axis. Central mechanisms include modulation of neurotrophic factors such as BDNF. They also involve regulation of immune cell function and inflammation, metabolic reprogramming, and gut-brain-muscle interactions. This multidimensional framework transcends traditional unifactorial models. It supports a unified “neuro-immune-metabolic axis” as the core mechanism underlying exercise-induced cognitive and psychological resilience. Crucially, recognizing the heterogeneity of exercise effects across populations and modalities is critical for developing personalized interventions. Future research integrating multi-omics technologies, longitudinal designs, and advanced imaging will be essential to unravel the precise molecular pathways and optimize exercise prescriptions for brain health across the lifespan.

## 2 Search strategy

Search strategy and selection criteria to ensure a comprehensive synthesis, we conducted a systematic search of PubMed, Web of Science, and Scopus databases for articles published up to December 2025. Search terms included combinations of “exercise,” “neuroinflammation,” “metabolism,” “kynurenine,” “microglia,” “gut-brain axis,” and “cognitive function.” We prioritized studies that (1) examined bidirectional cross-talk between at least two systems (neural, immune, metabolic); (2) provided mechanistic insights rather than purely descriptive outcomes; and (3) included high-quality randomized controlled trials (RCTs) or well-controlled animal models. Articles lacking full text or not published in English were excluded.

## 3 Exercise as an energy challenge: recalibration of central and peripheral metabolic communication

### 3.1 Metabolic signaling molecules induced by exercise and their neural actions

Exercise induces a profound metabolic challenge. It necessitates coordinated communication between peripheral tissues and the central nervous system (CNS) to maintain energy homeostasis. Key metabolites generated during exercise include lactate and ketone bodies. These serve as essential signaling molecules that traverse the blood–brain barrier (BBB) to modulate neuronal energy supply and function (Jang et al., 2023; Jensen et al., 2020; Plourde et al., 2024; Versele et al., 2020). Lactate is produced by active skeletal muscle through anaerobic glycolysis. It is transported into the brain via monocarboxylate transporters (MCTs) such as MCT1 and MCT4. Within the brain, lactate acts as an alternative fuel substrate for neurons and astrocytes. This support sustains synaptic activity and cognitive processes during and after exercise (Murphy et al., 2020). Similarly, ketone bodies including *β*-hydroxybutyrate form during prolonged or intense exercise. They cross the BBB and contribute to neuronal energy metabolism. This is particularly relevant when glucose availability is limited (Opialla et al., 2022). Beyond fuel provision, these metabolites function as signaling entities. They influence gene expression and neural plasticity in the CNS.

A central molecular mediator of exercise-induced neural adaptation is peroxisome proliferator-activated receptor gamma coactivator-1 alpha (PGC-1α). Exercise robustly upregulates PGC-1α expression in skeletal muscle. This upregulation in turn induces the expression of fibronectin type III domain-containing protein 5 (FNDC5) (Ali et al., 2024; Jo and Song, 2021; Pignataro et al., 2021). FNDC5 undergoes cleavage to release irisin, a myokine that enters the CNS. Irisin promotes the expression of BDNF. BDNF is a pivotal neurotrophin involved in synaptic plasticity and cognitive enhancement (Murphy et al., 2020; Zalouli et al., 2023). The PGC-1α/FNDC5/irisin axis thus acts as a molecular conduit. It links peripheral metabolic activity to central neural plasticity adaptations (Lee et al., 2025; Leger et al., 2024).

Beyond the PGC-1α-FNDC5-irisin axis, exercise-induced metabolic adaptations also exert neuroprotection through the regulation of the tryptophan-kynurenine pathway. Skeletal muscle PGC-1α upregulation stimulates the expression of kynurenine aminotransferases (KATs), enzymes that catalyze the peripheral conversion of kynurenine into kynurenic acid (KYNA). This conversion is critical because, unlike kynurenine, KYNA cannot cross the blood–brain barrier. By shifting the metabolic flux towards KYNA, exercise effectively reduces the availability of kynurenine for transport into the CNS, thereby preventing its central metabolism into the neurotoxic agonist quinolinic acid (QUIN). This mechanism functions as a “peripheral detoxification” system, shielding the brain from excitotoxicity and stress-induced inflammation, and provides a distinct metabolic link between muscle bioenergetics and psychological resilience (Agudelo et al., 2014; Allison et al., 2019).

Moreover, metabolic signals and neurotrophic factors such as BDNF exhibit synergistic regulation during exercise. BDNF expression is enhanced in response to metabolic changes. These changes include increased lactate and ketone levels. This enhancement facilitates synaptogenesis and remodeling of neural networks. Such networks are critical for learning and memory (Zalouli et al., 2023). This coordinated upregulation supports synapse formation and strengthening, thereby potentially facilitating the optimization of cognitive function. Collectively, these findings underscore the role of exercise-induced metabolites and myokines. They mediate neuroenergetic support and neural plasticity through integrated peripheral-central signaling pathways (Ajoy et al., 2021; Spanaki et al., 2024; Vezzoli et al., 2020) (Table 1).

**Table 1:** Core regulatory molecules in the neuro-immuno-metabolic (NIM) axis.

Molecule name	Core functions	Targets	Primary evidence source	References
Lactate	Neuronal energy substrate, BDNF regulation	Hippocampal neurons, MCT1/MCT4 transporters	Human & animal	Murphy et al. (2020), Plourde et al. (2024), and Vezzoli et al. (2020)
PGC-1α/irisin	Peripheral-central neural plasticity linkage	Skeletal muscle, central BDNF promoter	Animal (preclinical)	Ali et al. (2024), Lee et al. (2025), and Leger et al. (2024)
IL-6 (myokine)	IL-10 induction, repair-oriented inflammation initiation	Peripheral immune cells, microglia	Animal (preclinical)	Liu et al. (2024), Mao et al. (2020), and Stojanovic et al. (2025)
SCFAs (acetate/propionate/butyrate)	Neuroinflammation inhibition, neurogenesis promotion	Microglia, hypothalamic neurons	Animal (preclinical)	Caputi et al. (2021), Cintado et al. (2025), and Souza et al. (2023)
BDNF	Synaptic plasticity regulation, neuroprotection	Hippocampal/prefrontal TrkB receptors	Human & animal	Baranowski et al. (2023), Huang et al. (2021), and Oyovwi et al. (2025)

This table summarizes the associations between core regulatory molecules of the NIM axis, their biological functions, and target cells/tissues, highlighting the cross-system regulatory roles of metabolic, immune, and neural molecules. BDNF, brain-derived neurotrophic factor; MCT, monocarboxylate transporter; PGC-1α, peroxisome proliferator-activated receptor gamma coactivator-1 alpha; FNDC5, fibronectin type III domain-containing protein 5; IL, interleukin; SCFA, short-chain fatty acid.

### 3.2 Metabolic-immune cross-regulation mechanisms

Exercise-induced alterations in metabolic state profoundly influence immune cell function. This is especially true in the context of inter-organ communication between peripheral immune cells and the CNS. Systemic metabolic changes during and after exercise modulate immune cell activation. They also affect differentiation and trafficking of these cells. These changes in turn impact neuroimmune interactions (Arner and Rathmell, 2023; Hartmann et al., 2021). Metabolic reprogramming of immune cells is characterized by shifts in glycolytic and oxidative phosphorylation pathways. This reprogramming is essential for their functional adaptation (Hu et al., 2022). Exercise induces a metabolic milieu that favors anti-inflammatory immune phenotypes. It promotes the release of anti-inflammatory cytokines such as interleukin-10. This cytokine can cross the BBB or signal via peripheral nerves to modulate CNS inflammation (Ben-Khemis et al., 2023; Saxton et al., 2021; Yang et al., 2022).

**Figure 1:**
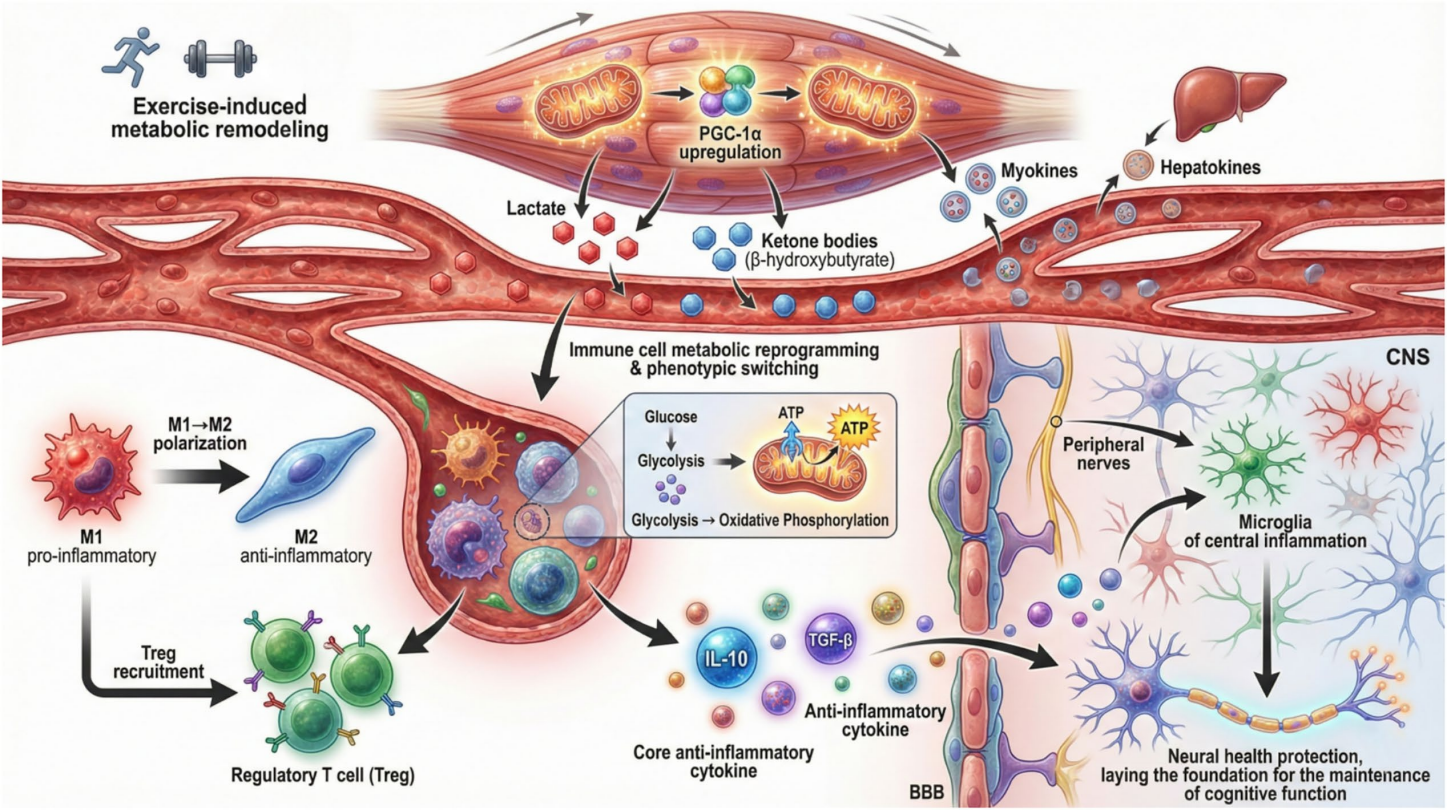
Metabolic-immune crosstalk in the NIM axis: exercise-induced reprogramming. Mechanistic model of exercise-induced metabolic-immune crosstalk in the NIM axis. Upstream, exercise triggers metabolic shifts (lactate elevation, ketone body production, PGC-1α upregulation). These metabolic signals drive immune cell metabolic reprogramming (switch from glycolysis to oxidative phosphorylation) and phenotypic conversion (macrophage M1 → M2 polarization). Consequently, anti-inflammatory cytokines (IL-10) are released, which modulate central neuroinflammation by crossing the blood–brain barrier or via peripheral nerve signaling. NIM, neuro-immuno-metabolic; PGC-1α, peroxisome proliferator-activated receptor gamma coactivator-1 alpha; IL, interleukin; BBB, blood–brain barrier.

Furthermore, exercise-induced metabolic adaptations facilitate peripheral immune signal communication to the CNS. For example, metabolites such as lactate and ketone bodies influence immune cell metabolism. They also regulate cytokine production by these cells. This shaping of systemic inflammatory responses contributes to neural health (Duraj et al., 2024; Watson et al., 2023). The release of myokines and hepatokines during exercise also modulates immune function. It enhances anti-inflammatory pathways and promotes immune tolerance (Babaei and Yadegari, 2025). This metabolic-immune crosstalk is critical for maintaining CNS homeostasis. It may underlie the beneficial effects of exercise on neuroinflammatory and neurodegenerative conditions. Thus, exercise-induced metabolic shifts act as key regulators of immune cell function. They also modulate immune signaling to the CNS, fostering an anti-inflammatory environment conducive to neural health (Figure 1).

### 3.3 Dynamic bidirectional communication of the neuro-metabolic axis

The CNS possesses sophisticated mechanisms to sense peripheral metabolic changes. It also responds to these changes to orchestrate energy distribution and neural activity. This optimization of physiological function occurs during exercise. Central metabolic sensing involves specialized neurons in hypothalamic and brainstem nuclei. These neurons detect circulating nutrients, hormones, and metabolites. They adjust autonomic outputs accordingly (Lin et al., 2025; Ma et al., 2023). Exercise induces temporal and spatial coordination of energy metabolism and neural activity. This ensures cognitive functions are maintained or enhanced. The coordination persists despite increased systemic energy demands (Dang et al., 2024; Lao-Peregrin et al., 2024; Yang et al., 2023). During exercise, the CNS integrates metabolic signals such as glucose, lactate, and ketones. It modulates neuronal excitability and synaptic plasticity. This modulation supports motor and cognitive performance (Dejanovic et al., 2024; Lao-Peregrin et al., 2024). Dynamic changes in cerebral blood flow and substrate utilization facilitate this integration. These changes are tightly regulated to meet fluctuating energy requirements of active neural circuits (Krolak et al., 2025; Oprea et al., 2025). Additionally, the CNS modulates peripheral metabolism through autonomic nervous system outputs. It influences substrate mobilization, insulin sensitivity, and inflammatory status. This creates a feedback loop that sustains metabolic homeostasis (Actis Dato et al., 2024; Tucci et al., 2021; Wan et al., 2025). The temporal coordination of metabolic and neural responses during exercise is critical for optimizing cognitive outcomes. Evidence indicates that metabolic flexibility and efficient substrate switching underpin enhanced cognitive resilience (Ang et al., 2025; Grasmann et al., 2021; Tsilingiris et al., 2021). This bidirectional neuro-metabolic communication axis exemplifies integrated physiological adaptations to exercise (Table 2).

**Table 2:** Core signaling pathways in the neuro-immuno-metabolic (NIM) axis.

Signaling pathway	Core molecular components	Main biological functions	Primary evidence source	References
PGC-1α/FNDC5/irisin/BDNF	PGC-1α, FNDC5, irisin, BDNF	Metabolic-neural cross-talk; promote neurogenesis, synaptic plasticity	Animal (preclinical)	Ali et al. (2024), Lee et al. (2025), and Zalouli et al. (2023)
AMPK-PGC1α-PPARγ	AMPK, PGC1α, PPARγ	Immune cell metabolic reprogramming; induce anti-inflammatory phenotype	Animal (preclinical)	Hu et al. (2022), Murugathasan et al. (2023), and Ying et al. (2025)
IL-6-IL-10-microglial polarization	IL-6 (myokine), IL-10, microglia	Initiate repair-oriented inflammation; inhibit neuroinflammation	Animal (preclinical)	Liu et al. (2021), Mao et al. (2020), and Yu and Chen (2025)
SCFA-TLR4-cytokine	SCFAs, TLR4, anti-inflammatory cytokines	Protect intestinal barrier; alleviate central neuroinflammation	Animal (preclinical)	Caputi et al. (2021), Dmytriv et al. (2024), and Souza et al. (2023)

This table illustrates the associations between key signaling pathways of the NIM axis, their core molecular components, and cross-system regulatory functions, highlighting the integrated mechanism of exercise-induced brain health benefits. NIM, neuro-immuno-metabolic; PGC-1α, peroxisome proliferator-activated receptor gamma coactivator-1 alpha; FNDC5, fibronectin type III domain-containing protein 5; BDNF, brain-derived neurotrophic factor; AMPK, AMP-activated protein kinase; PPARγ, peroxisome proliferator-activated receptor gamma; IL, interleukin; SCFA, short-chain fatty acid; TLR4, Toll-like receptor 4.

## 4 Exercise-induced remodeling of the neuroimmune axis: a paradigm shift from pathological inflammation to reparative programs

### 4.1 Limitations of traditional concepts of neuroinflammation

Traditional views of neuroinflammation have predominantly characterized inflammation as a pathological process. It is detrimental to neural function and often implicated as a primary driver of neurodegenerative diseases and cognitive impairment (Moujalled et al., 2021; Tian et al., 2022). This perspective tends to frame CNS inflammation as a uniform, deleterious response. It leads to neuronal damage and dysfunction. However, such a unidimensional understanding neglects key features of CNS inflammatory processes. These include inherent complexity, diversity, and temporal dynamics (Adamu et al., 2024; Li et al., 2024). In reality, neuroinflammation encompasses a spectrum of responses. These can be harmful or beneficial depending on context, intensity, and activation stage. Current evidence suggests that microglia do not merely switch between binary “M1” (neurotoxic) and “M2” (neuroprotective) states, but rather exist along a dynamic multidimensional spectrum of activation. Depending on the metabolic and environmental context, microglia can adopt specific transcriptional signatures that range from pro-inflammatory surveillance to repair-oriented functional states. These states are temporally regulated to support tissue remodeling and synaptic plasticity rather than simply suppressing inflammation (Faust et al., 2025; Guo et al., 2022; Wang et al., 2021; Yu et al., 2023). Exercise-induced inflammatory responses exemplify this duality. Unlike pathological inflammation linked to chronic neurodegeneration, exercise triggers a transient regulated inflammatory milieu. This milieu promotes neuroprotection and repair. Acute exercise bouts induce controlled elevations in cytokines such as interleukin-6 (IL-6). In this context, IL-6 acts as a myokine with systemic anti-inflammatory effects. It does not function as a pro-inflammatory mediator (Mao et al., 2020; Ren et al., 2020; Zheng et al., 2020). Furthermore, exercise modulates immune cell populations and their activation states. It enhances recruitment and function of regulatory immune cells. These cells mitigate chronic inflammation. This nuanced understanding challenges the traditional inflammation paradigm. It highlights that inflammation is not inherently pathological. Instead, it can be a critical component of CNS homeostasis and recovery (Dikiy and Rudensky, 2023; Rogovskii, 2020). Therefore, distinguishing between pathological and physiological inflammation is essential. Physiological inflammation includes exercise-induced responses (Table 3). This distinction aids in developing targeted interventions. These interventions harness beneficial neuroimmune interactions while mitigating harmful ones (Barad et al., 2023; Su and Su, 2025).

**Table 3:** Pathological vs. exercise-induced repair-oriented inflammation.

Characteristic	Pathological inflammation	Repair-oriented inflammation	Primary evidence source	References
Immune cell phenotype	Microglial M1 polarization; pro-inflammatory cytokine dominance	Microglial M1 to M2 polarization; Treg recruitment	Animal (preclinical)	Guo et al. (2022), Mao et al. (2020), and Wang et al. (2021)
Key molecules	Increased TNF-α, IL-1β; pro-inflammatory chemokines	Increased IL-6 (myokine), IL-10, BDNF; reduced pro-inflammatory cytokines	Animal (preclinical)	Liu et al. (2021), Murugathasan et al. (2023), and Yu and Chen (2025)
Neural effects	Synaptic damage, neuronal dysfunction; cognitive decline	Synaptic remodeling, neurogenesis; neuronal protection, debris clearance	Animal (preclinical)	Nicastri et al. (2022), Yu and Chen (2025), and Zalouli et al. (2023)
Molecular mechanism	Sustained NF-κB activation; oxidative stress, mitochondrial DNA release	AMPK-PGC1α-PPARγ activation; enhanced mitochondrial function	Animal (preclinical)	Hu et al. (2025), Liu et al. (2024), and Ying et al. (2025)

This table compares the characteristics of pathological inflammation and exercise-induced repair-oriented inflammation in terms of immune phenotype, key molecules, neural effects, and molecular mechanisms, highlighting the dual nature of neuroinflammation. TNF-α, tumor necrosis factor alpha; IL, interleukin; BDNF, brain-derived neurotrophic factor; NF-κB, nuclear factor kappa B; AMPK, AMP-activated protein kinase; PGC-1α, peroxisome proliferator-activated receptor gamma coactivator-1 alpha; PPARγ, peroxisome proliferator-activated receptor gamma.

### 4.2 Exercise-induced reparative inflammatory mechanisms

Exercise initiates a complex reparative inflammatory cascade. This cascade orchestrates systemic and central immune responses. These responses are conducive to neural repair and cognitive enhancement. A pivotal mediator in this process is interleukin-6 (IL-6). It is released from contracting skeletal muscles during exercise. IL-6 exhibits a dual role. It acts as both a pro-inflammatory cytokine and an anti-inflammatory myokine (Liu et al., 2024; Raut and Cucullo, 2025; Stojanovic et al., 2025). Exercise-induced IL-6 elevation transiently stimulates anti-inflammatory pathways. This includes upregulation of interleukin-10 (IL-10). IL-10 is an anti-inflammatory cytokine. It plays a critical role in modulating microglial activation. It also promotes a reparative phenotype (Illes et al., 2020; Pang et al., 2022; Radin and Tsirka, 2020). IL-10 upregulation in the CNS contributes to microglial remodeling. It shifts microglia from a pro-inflammatory to an anti-inflammatory state. This fosters an environment conducive to neuroprotection and synaptic plasticity. Concurrently, exercise activates neuroprotective programs. These include enhanced antioxidant defenses. They also involve increased expression of neurotrophic factors such as BDNF (Lu et al., 2025; Malange et al., 2022). These neurotrophic factors support neuronal survival. They promote neurogenesis and facilitate synaptic remodeling. Collectively, these effects underpin cognitive improvements.

**Figure 2:**
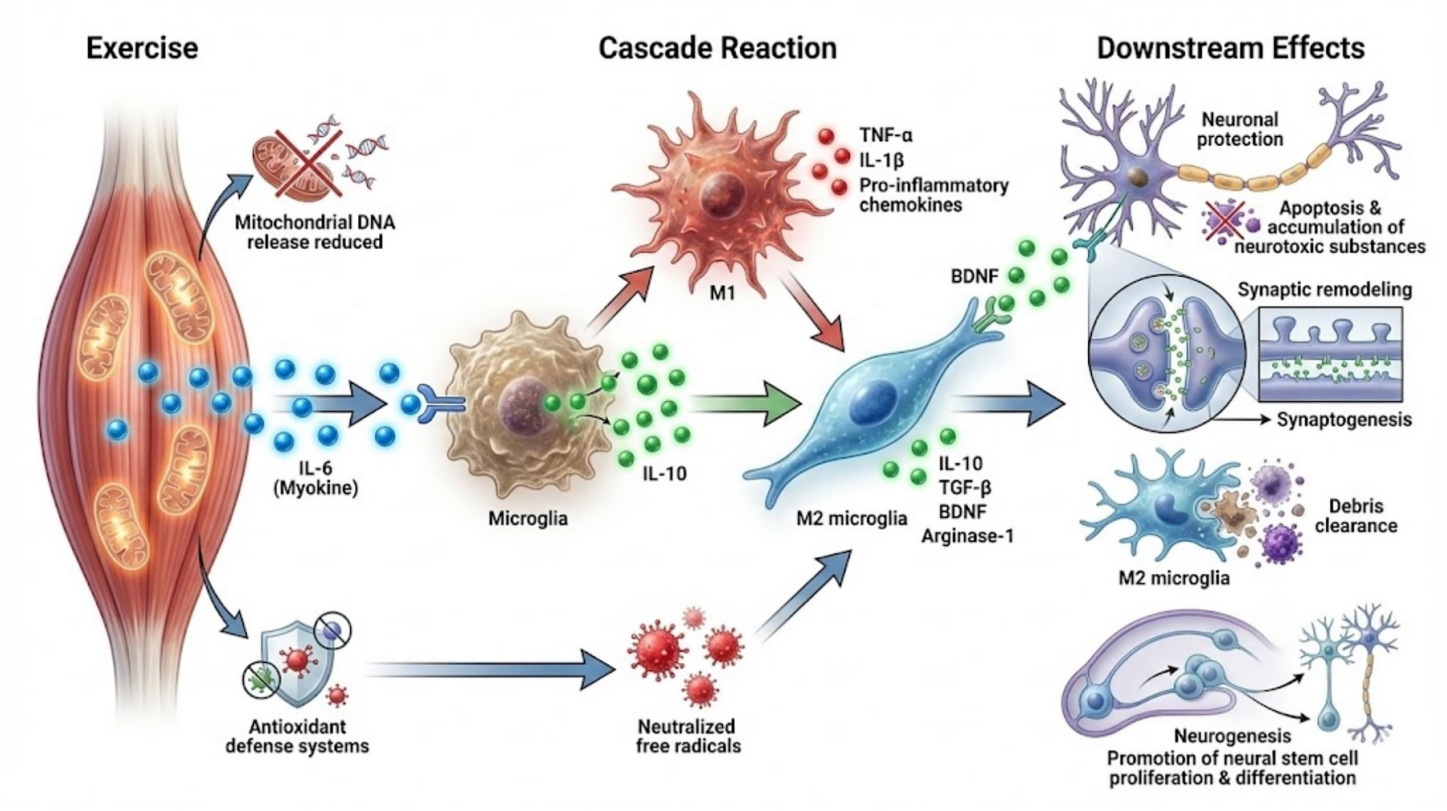
Exercise-induced resolving inflammation: a neuroprotective cascade. Schematic of the exercise-induced reparative inflammatory cascade. Skeletal muscle contraction during exercise releases IL-6 as a myokine, initiating a sequential anti-inflammatory program: IL-6 upregulates IL-10 expression, which drives microglial phenotypic shifts from a pro-inflammatory state toward a resolving, reparative phenotype (characterized by upregulation of markers such as CD206 and TGF-*β*, and metabolic reprogramming toward oxidative phosphorylation). IL, interleukin; M1, classically activated macrophage phenotype; M2, alternatively activated macrophage phenotype; TGF-β, transforming growth factor beta; BDNF, brain-derived neurotrophic factor.

Additionally, exercise-induced improvements in mitochondrial function reduce oxidative stress. They also decrease mitochondrial DNA release. Such release can otherwise trigger innate immune activation and neuroinflammation (Benedict and Joshi, 2025; Gong et al., 2024; Zhang et al., 2025). Modulation of mitochondrial quality and metabolic pathways in immune cells further tempers inflammatory responses. Macrophages are a key example of such immune cells. This modulation supports tissue repair. This multifaceted reparative inflammatory mechanism underscores a key synergy. Exercise-induced cytokine signaling and metabolic adaptations work together. They remodel the CNS immune landscape. This shift moves from chronic inflammation to a state favoring repair and resilience (Barad et al., 2023; Seo et al., 2019; Wen et al., 2025) (Figure 2).

### 4.3 Dynamic regulation of immune cell subtypes and neural function by exercise

Exercise induces dynamic alterations in immune cell subtypes. These changes occur both in the periphery and within the CNS. They critically influence neural plasticity and function. Within the CNS, microglia are the resident immune cells. They undergo exercise-induced phenotypic shifts. These shifts are characterized by reduced pro-inflammatory markers. They also involve increased anti-inflammatory and neuroprotective profiles (Chen et al., 2023; Ronaldson and Davis, 2020; Zhang et al., 2021). This functional shift towards a reparative phenotype enhances microglial capacities. These include debris clearance, secretion of neurotrophic factors, and support of synaptic remodeling. These capacities facilitate neuroplasticity and cognitive resilience. Peripheral immune cells also participate in this dynamic regulation. Exercise promotes mobilization and trafficking of lymphocytes. Regulatory T cells (Tregs) are a key subset of these lymphocytes. They can infiltrate the CNS and modulate local immune environments (Deng et al., 2023; Qu et al., 2024; Zhang et al., 2025). Crosstalk between peripheral immune cells and CNS-resident cells is further influenced by exercise. It alters blood–brain barrier permeability and chemokine expression. These alterations regulate immune cell migration (Hu et al., 2025).

Notably, recruitment of regulatory immune subsets helps suppress chronic neuroinflammation. It also supports repair processes. Moreover, exercise modulates the balance of immune cell subpopulations. It increases naïve T cells and reduces senescent or exhausted phenotypes. This is particularly relevant in aging populations. Immune senescence in these populations contributes to neurodegenerative risk. These immune cell dynamics link to key cellular improvements. They are associated with enhanced mitochondrial function. They also involve metabolic reprogramming within immune cells. These changes further boost their reparative capacities (Lewis et al., 2025; Rocamora-Reverte et al., 2022; Yin et al., 2025). Collectively, exercise-induced remodeling of immune cell subtypes is pivotal. Their interactions with neural cells form a key mechanism. This mechanism provides a plausible pathway by which physical activity promotes CNS repair, supporting cognitive enhancement and psychological resilience (Mu et al., 2025; Papp et al., 2021; Wang et al., 2022). However, the precise molecular signatures characterizing these immune shifts remain to be fully mapped, underscoring the need for advanced single-cell sequencing approaches.

## 5 Exercise as a modulator of the muscle-gut-brain axis: neuroimmune integration via the microbiome and metabolites

### 5.1 Gut microbiota composition and exercise-related changes

Exercise is increasingly recognized as a key modulator of gut microbiota composition, diversity, and abundance. These microbial changes in turn influence systemic health and brain function. Regular physical activity promotes increased microbial diversity. It also fosters the proliferation of beneficial bacterial taxa, particularly those within the Firmicutes phylum. This phylum is often linked to positive health outcomes (Dalton et al., 2019). Studies in humans and animal models have yielded consistent findings. Endurance and moderate-intensity aerobic exercise enhance the relative abundance of genera such as Bifidobacterium, Blautia, and Phascolarctobacterium. These bacteria contribute to improved metabolic and immune functions (Aragón-Vela et al., 2021; Yang et al., 2021). Conversely, sedentary lifestyles correlate with reduced microbial diversity and dysbiosis. These conditions are associated with metabolic and neuropsychiatric disorders. Exercise-induced shifts in gut microbiota vary across exercise modalities. For example, cardiorespiratory exercise induces transient alterations in microbiome composition. Resistance training may exert minimal effects on microbial structure (Bycura et al., 2021).

Beyond compositional changes, exercise influences the functional capacity of the microbiome. It enhances pathways related to short-chain fatty acid (SCFA) metabolism and amino acid catabolism. These pathways are critical for host energy homeostasis and immune regulation (Chen See et al., 2022; Greenhill, 2020). Importantly, exercise modulates intestinal barrier integrity. Evidence includes increased expression of tight junction proteins such as ZO-1, Occludin, and Claudin-1. This upregulation reduces intestinal permeability and systemic endotoxemia (Santos et al., 2024; Segui-Perez et al., 2024; Yu et al., 2017). Improved gut barrier function mitigates chronic low-grade inflammation. Such inflammation can adversely affect brain health. Moreover, exercise-induced microbial changes influence systemic immune status. They modulate pro- and anti-inflammatory cytokine profiles, contributing to a balanced immune response (Quaresma et al., 2024). Collectively, these findings underscore that exercise reshapes the gut microbiota ecosystem. It enhances microbial diversity, promotes beneficial bacteria, and strengthens gut barrier function. These adaptations together support systemic immune homeostasis. They potentially mediate exercise-induced cognitive and psychological benefits (Table 4).

**Table 4:** Gut microbiota-metabolite-neural effect correlation.

Dominant microbiota	Core metabolites	Neural effects	Primary evidence source	References
Firmicutes phylum	Short-chain fatty acids (SCFAs)	Promote neurogenesis; increase BDNF, inhibit neuroinflammation	Human & animal	Aragón-Vela et al. (2021), Cintado et al. (2025), and Quaresma et al. (2024)
Bifidobacterium genus	Acetate, propionate, tryptophan metabolites	Regulate emotion; anxiolytic effect, modulate CNS serotonin	Animal (preclinical)	Dalton et al. (2019), Molska et al. (2024), and Yang et al. (2021)
Blautia genus	Butyrate, amino acid derivatives	Enhance cognitive flexibility; consolidate memory	Human (observational) & animal	Aragón-Vela et al. (2021), Bycura et al. (2021), and Okamoto et al. (2019)
Phascolarctobacterium genus	SCFAs, 3-Hydroxyphenylacetic acid	Enhance stress resistance, psychological resilience	Animal (preclinical)	Chen See et al. (2022), Greenhill (2020), and Yang et al. (2021)

This table summarizes the associations between exercise-enriched gut microbiota, their core metabolites, and corresponding neural effects, highlighting the mediating role of the gut microbiota-metabolite axis in the NIM axis. NIM, neuro-immuno-metabolic; SCFA, short-chain fatty acid; BDNF, brain-derived neurotrophic factor; CNS, central nervous system.

### 5.2 Role of microbial metabolites in the neuro-immune-metabolic axis

Microbial metabolites, particularly short-chain fatty acids (SCFAs) like acetate, propionate, and butyrate, act as crucial mediators. They link the gut microbiota to the central nervous system (CNS) via the neuro-immune-metabolic axis. These SCFAs are produced through gut bacterial fermentation of dietary fibers. They exert systemic effects extending to brain function and immune modulation (Erny et al., 2021; Hays et al., 2024; Portincasa et al., 2022). SCFAs influence brain health through multiple specific mechanisms. They can cross the blood–brain barrier or signal via the vagus nerve. This modulation affects neuroinflammation, neuroplasticity, and neurotransmitter synthesis (Caputi et al., 2021; Shin et al., 2019). For instance, butyrate functions as a histone deacetylase inhibitor. It promotes gene expression that supports neurogenesis and synaptic plasticity. These processes are essential for cognitive function and mood regulation. Additionally, SCFAs regulate central immune cells such as microglia. They attenuate microglial activation and reduce neuroinflammation. Neuroinflammation is a key factor in neurodegenerative and psychiatric disorders (Zhang et al., 2022).

**Figure 3:**
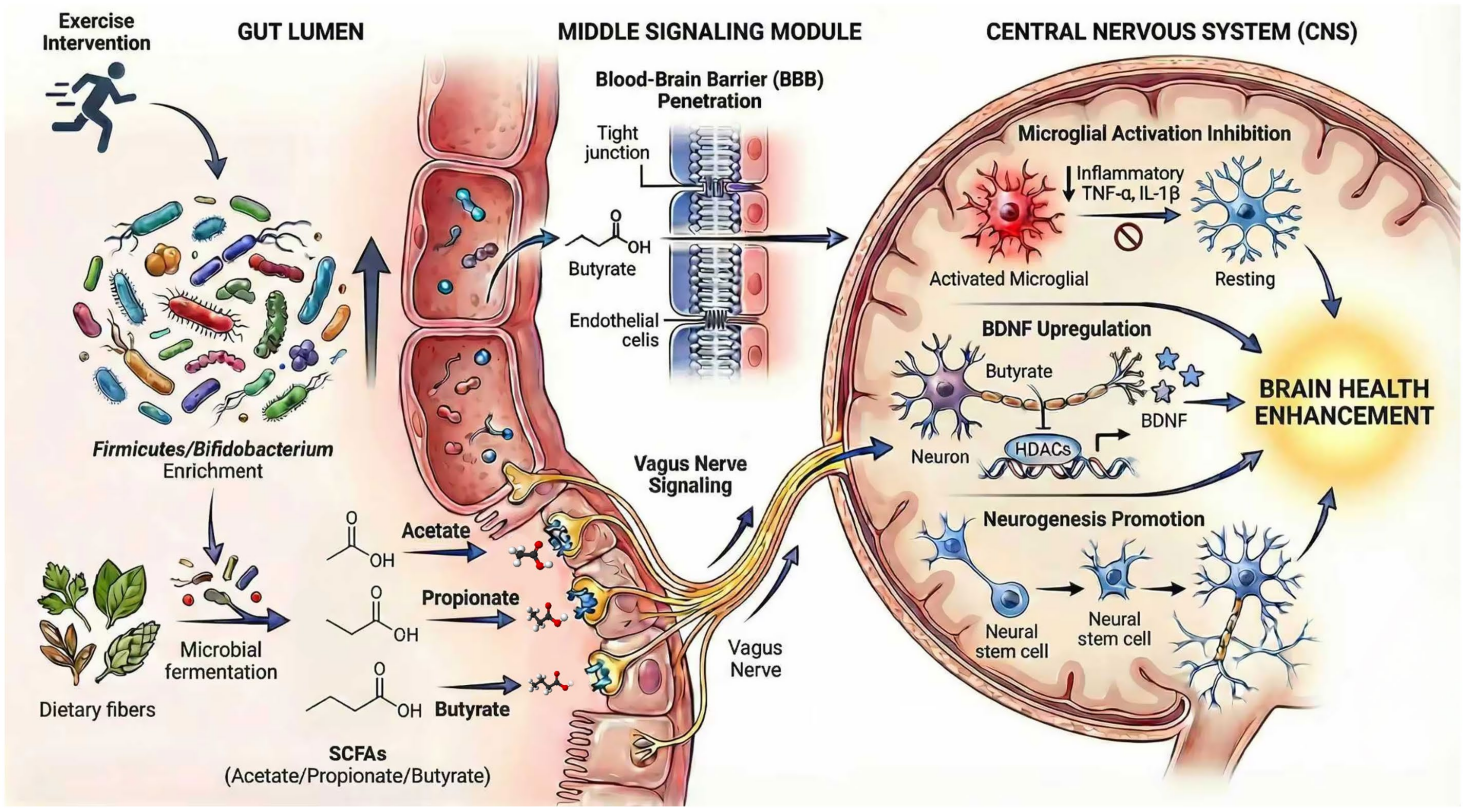
Microbial metabolites as messengers in the gut-brain axis: exercise-modulated pathways. Mechanisms of microbial metabolites mediating gut-brain communication in exercise. Left, exercise remodels gut microbiota (enrichment of Firmicutes phylum, Bifidobacterium genus, etc.) to enhance production of short-chain fatty acids (SCFAs: acetate, propionate, butyrate) and secondary metabolites (tryptophan metabolites). Middle, SCFAs transmit signals to the central nervous system (CNS) via two pathways: direct penetration of the blood–brain barrier (BBB) and indirect signaling through the vagus nerve. Right, these metabolites exert central effects including inhibiting microglial activation, upregulating BDNF expression, promoting neurogenesis in the hippocampal dentate gyrus, and modulating neurotransmitter (serotonin) synthesis. All adaptations converge to improve brain health. SCFA, short-chain fatty acid; CNS, central nervous system; BBB, blood–brain barrier; BDNF, brain-derived neurotrophic factor.

Beyond SCFAs, other microbial metabolites have notable effects. Compounds like 3-Hydroxyphenylacetic acid and 4-Hydroxybenzoic acid confer cardioprotective effects post-myocardial infarction. This highlights the systemic reach of microbial metabolites (Zhou et al., 2022). Crucially, the gut microbiota metabolizes dietary tryptophan into indole and its derivatives (indole-3-propionic acid). These metabolites function as endogenous agonists for the aryl hydrocarbon receptor (AhR) on astrocytes and microglia. Activation of AhR signaling promotes the differentiation of regulatory T cells (Tregs) and suppresses neuroinflammation, thereby linking gut microbial metabolism directly to CNS immune tolerance and providing a complementary pathway to SCFAs for neuroprotection (Kiran et al., 2025). The interplay between microbial metabolites and host immune signaling pathways further integrates gut-derived signals. Key pathways include TLR4 and cytokine cascades. These interactions shape systemic immune responses that impact brain function (Dmytriv et al., 2024; Jing et al., 2025). Notably, exercise enhances SCFA production by promoting SCFA-producing bacteria. This amplification strengthens neuro-immune-metabolic effects. It contributes to improved exercise capacity, metabolic efficiency, and psychological resilience. Therefore, microbial metabolites act as pivotal biochemical messengers within the neuro-immune-metabolic axis, likely serving as key mediators of the beneficial effects of exercise on brain health and systemic immunity (Qiao et al., 2025; Zhao et al., 2025) (Figure 3).

### 5.3 Integrative perspective of the gut-brain and muscle-brain axes

The gut-brain axis and muscle-brain axis are interconnected communication networks. They collectively form a comprehensive neuro-immune-metabolic framework. This framework mediates exercise-induced benefits on brain function and psychological resilience. The gut-brain axis involves bidirectional signaling between the gastrointestinal tract and the CNS. Neural pathways such as the vagus nerve, endocrine, immune, and metabolic routes mediate this communication. The gut microbiota plays a central role in this axis (Thaiss, 2023; Xia and Huang, 2025). Concurrently, skeletal muscle functions as an endocrine organ. It secretes myokines and exerkines such as irisin and BDNF. These factors influence neuroplasticity, neurogenesis, and systemic immune responses (Cutuli et al., 2023; Saponaro et al., 2024). Exercise modulates both axes synergistically. It remodels gut microbiota composition and function. This enhancement increases production of metabolites like SCFAs that influence muscle metabolism and brain function. Meanwhile, muscle-derived factors reciprocally affect gut physiology and microbiota composition (Scriven et al., 2023; Yin et al., 2022).

**Figure 4:**
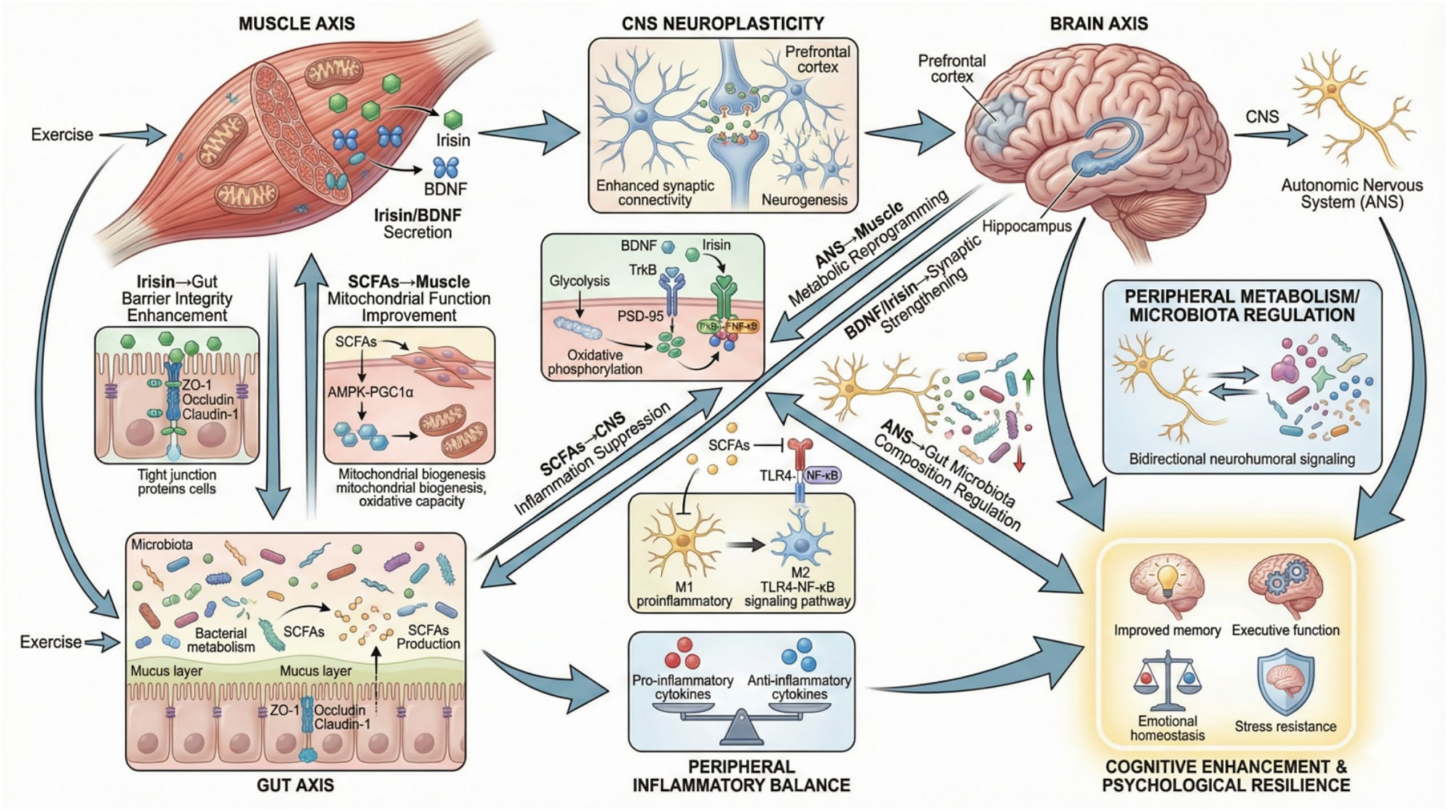
Muscle-gut-brain axis: bidirectional communication in exercise-induced brain health. Schematic of the muscle-gut-brain axis’s triangular bidirectional network. It comprises three core axes (muscle/gut/brain) with key signaling paths and inter-axis crosstalk, ultimately boosting cognitive enhancement and psychological resilience, visualizing the NIM axis’s integrative interactions. NIM, neuro-immuno-metabolic.

This bidirectional crosstalk establishes a muscle-gut-brain network. It integrates metabolic, immune, and neuroendocrine signals to optimize cognitive performance and psychological well-being (Cammisuli et al., 2022). For example, exercise-induced increases in irisin promote hippocampal BDNF expression. This supports cognitive enhancement. Gut microbiota-derived metabolites modulate systemic inflammation and neurotransmitter systems. These systems are involved in mood regulation (Molska et al., 2024). Furthermore, the gut microbiome influences exercise motivation. It acts through endocannabinoid-mediated pathways that enhance dopaminergic signaling in the brain. This illustrates a microbiome-dependent gut-brain-muscle interaction (Dohnalová et al., 2022; Sun et al., 2023). The integration of these axes also involves immune modulation. Exercise-induced changes in gut and muscle secretions regulate central and peripheral immune cells. This contributes to neuroprotection and stress resilience (Cammisuli et al., 2022; Schlegel et al., 2019). Collectively, the gut-brain and muscle-brain axes coalesce into a unified neuro-immune-metabolic network. Exercise-induced modulation of gut microbiota and muscle-derived factors orchestrates systemic and central adaptations. These adaptations enhance brain function and psychological resilience. This integrative perspective underscores the therapeutic potential of targeting the muscle-gut-brain axis. It aims to optimize cognitive health and mental well-being (Bower and Kuhlman, 2023) (Figure 4).

## 6 Conclusions and perspectives

In conclusion, this review has systematically constructed a unified framework of the neuro-immune-metabolic (NIM) axis. It marks a notable advancement beyond traditional isolated mechanistic views. Exercise serves as a dynamic energy challenge that integrates neural, immune, and metabolic systems, clarifying how physical activity enhances cognitive function and psychological resilience. This holistic approach underscores the need to move beyond siloed research paradigms, helping to appreciate the complex bidirectional interactions underpinning exercise-induced neurobiological benefits.

Notably, the NIM axis presented in this review is distinct from existing frameworks. While models such as the muscle-brain axis, gut-brain axis, psychoneuroimmunology, and immunometabolism have independently elucidated key bidirectional pathways, they often examine physiological interactions in isolation or focus primarily on pathological states. The NIM axis represents a fundamental shift towards a unified, tri-directional regulatory network. Unlike psychoneuroimmunology, which traditionally emphasizes stress-induced immune modulation, or immunometabolism, which focuses on cellular energetics, this framework positions physical exercise as a systemic “energy challenge” that forces a recalibration across all three systems simultaneously. Its distinctive novelty lies in the identification of “repair-oriented inflammation” as the central integrator—where metabolic signals (e.g., lactate, ketones) act as the trigger to switch immune phenotypes from pathological to reparative, directly driving neural plasticity. Thus, the NIM axis offers a mechanistic explanation for how metabolic stress is transduced into psychological resilience, a dynamic often overlooked in linear bipartite models.

A particularly transformative insight discussed herein is the concept of exercise-induced “resolving inflammation.” It challenges the long-standing dogma that inflammation is solely detrimental in the nervous system. Instead, this review highlights inflammation’s dual nature, emphasizing its critical role in neuroprotection and repair. This nuanced understanding prompts a reevaluation of neuroinflammatory processes in the context of exercise and neurological health, suggesting controlled inflammatory responses may be harnessed therapeutically to promote brain resilience and recovery.

Furthermore, the deep integration of the gut-brain and muscle-brain axes within the NIM framework reveals key insights. It identifies the microbiome and its metabolites as central players in exercise neurobiology. This intersection not only broadens the scope of exercise neuroscience but also bridges metabolic and immune pathways with neural function, positioning the microbiota as a pivotal mediator of exercise’s systemic effects. Such insights encourage a multidisciplinary research approach that combines microbiology, immunology, metabolism, and neuroscience to unravel complex crosstalk.

Notably, this review delineates the mechanistic specificity of different exercise modalities, clarifying how they activate the NIM axis (Table 5) and lay a solid theoretical foundation for precision exercise medicine. Recognizing that specific exercise modalities elicit distinct neuro-immune-metabolic responses enables tailored interventions aimed at optimizing cognitive and mental health outcomes. This precision approach holds promise for addressing heterogeneity in exercise responsiveness, particularly for individuals who show minimal or no benefit from conventional exercise regimens.

**Table 5:** Mechanistic specificity of exercise modalities in the NIM axis.

Exercise modality	Primary metabolic/immune mediators	Key neural mechanisms	Cognitive & mental health outcomes	Primary evidence source	References
Aerobic training (AT)	PGC-1α, Irisin, VEGF, BDNF (chronic elevation)	Hippocampal neurogenesis; Increased cerebral blood flow; Synaptic angiogenesis	Enhanced memory and learning; Reduced depression/anxiety; Increased gray matter volume	Human RCTs & animal models	Antunes et al. (2020), Balbim et al. (2024), and Enette et al. (2020)
Resistance training (RT)	IGF-1, Myostatin inhibition, IL-6 (myokine), Lactate (moderate)	White matter integrity (myelination); Frontal lobe activation; Functional connectivity	Improved executive function; Attention control; Reduced cognitive frailty	Human RCTs (limited animal data)	Balbim et al. (2024) and Herbert et al. (2020)
High-intensity interval training (HIIT)	Lactate (high surge), Ketone bodies, BDNF (acute spike), Catecholamines	Cortical excitability; “Energy challenge” induced plasticity; Rapid metabolic reprogramming	Cognitive flexibility; Inhibitory control; Rapid mood elevation	Human RCTs & animal models	Dos Santos et al. (2020) and Waddington et al. (2024)

This table delineates the distinct signaling pathways and neural outcomes associated with different exercise modalities, highlighting the necessity of precision exercise prescriptions. Bold text indicates the dominant mediators for each modality. NIM, neuro-immuno-metabolic; AT, aerobic training; RT, resistance training; HIIT, high-intensity interval training; PGC-1α, peroxisome proliferator-activated receptor gamma coactivator-1 alpha; VEGF, vascular endothelial growth factor; BDNF, brain-derived neurotrophic factor; IGF-1, insulin-like growth factor 1; IL, interleukin.

Looking forward, advancing this field will require leveraging cutting-edge technologies. Key examples include multi-omics, single-cell sequencing, and advanced neuroimaging. These tools will be indispensable for dissecting intricate cellular and molecular mechanisms that drive exercise-induced neuroplasticity and immune modulation. Furthermore, they will facilitate the development of mechanism-driven individualized exercise interventions that can overcome the challenge of non-responders and maximize therapeutic efficacy (Wang et al., 2024; Zhou et al., 2025).

Additionally, future studies must account for confounding lifestyle variables. Factors such as diet, sleep quality, and stress management likely interact with exercise to synergistically or antagonistically modulate the NIM axis.

In synthesizing diverse research perspectives and findings, this review advocates for a balanced integrative research paradigm. It appreciates both the complexity and specificity of exercise-induced neuro-immune-metabolic interactions. The convergence of these systems not only enriches our understanding of exercise neuroscience but also opens new avenues for clinical translation. Ultimately, this unified NIM axis framework heralds a new era in exercise neurobiology, promising to revolutionize how we harness physical activity to enhance brain health and psychological resilience across the lifespan.

## References

[ref1] Actis Dato V. Lange S. Cho Y. (2024). Metabolic flexibility of the heart: the role of fatty acid metabolism in health, heart failure, and cardiometabolic diseases. Int. J. Mol. Sci. 25:1211. doi: 10.3390/ijms25021211, 38279217 PMC10816475

[ref2] Adamu A. Li S. Gao F. Xue G. (2024). The role of neuroinflammation in neurodegenerative diseases: current understanding and future therapeutic targets. Front. Aging Neurosci. 16:1347987. doi: 10.3389/fnagi.2024.1347987, 38681666 PMC11045904

[ref3] Ajoy R. Lo Y.-C. Ho M.-H. Chen Y.-Y. Wang Y. Chen Y.-H. . (2021). CCL5 promotion of bioenergy metabolism is crucial for hippocampal synapse complex and memory formation. Mol. Psychiatry 26, 6451–6468. doi: 10.1038/s41380-021-01103-3, 33931731 PMC8760051

[ref9001] Agudelo L. Z. Femenía T. Orhan F. Porsmyr-Palmertz M. Goiny M. Martinez-Redondo V. . (2014). Skeletal muscle PGC-1α1 modulates kynurenine metabolism and mediates resilience to stress-induced depression. Cell, 159, 33–45. doi: 10.1016/j.cell.2014.07.051 25259918

[ref4] Ali N. H. Alhamdan N. A. Al-Kuraishy H. M. Al-Gareeb A. I. Elekhnawy E. Batiha G. E.-S. (2024). Irisin/PGC-1α/FNDC5 pathway in Parkinson’s disease: truth under the throes. Naunyn Schmiedeberg's Arch. Pharmacol. 397, 1985–1995. doi: 10.1007/s00210-023-02726-9, 37819389

[ref9002] Allison D. J. Nederveen J. P. Snijders T. Bell K. E. Kumbhare D. Phillips S. M. . (2019). Exercise training impacts skeletal muscle gene expression related to the kynurenine pathway. Am J Physiol Cell Physiol, 316, C444–c448. doi: 10.1152/ajpcell.00448.2018 30649918 PMC6457098

[ref5] Ang J. C. Sun L. Foo S. R. Leow M. K. Vidal-Puig A. Fontana L. . (2025). Perspectives on whole body and tissue-specific metabolic flexibility and implications in cardiometabolic diseases. Cell Rep. Med. 6:102354. doi: 10.1016/j.xcrm.2025.102354, 40961926 PMC12490259

[ref6] Antunes B. M. Rossi F. E. Teixeira A. M. Lira F. S. (2020). Short-time high-intensity exercise increases peripheral BDNF in a physical fitness-dependent way in healthy men. Eur. J. Sport Sci. 20, 43–50. doi: 10.1080/17461391.2019.1611929, 31057094

[ref7] Aragón-Vela J. Solis-Urra P. Ruiz-Ojeda F. J. Álvarez-Mercado A. I. Olivares-Arancibia J. Plaza-Diaz J. (2021). Impact of exercise on gut microbiota in obesity. Nutrients 13:3999. doi: 10.3390/nu13113999, 34836254 PMC8624603

[ref8] Arner E. N. Rathmell J. C. (2023). Metabolic programming and immune suppression in the tumor microenvironment. Cancer Cell 41, 421–433. doi: 10.1016/j.ccell.2023.01.009, 36801000 PMC10023409

[ref9] Babaei P. Yadegari F. (2025). How exercise affects Exerkines in metabolic syndrome. Horm. Metab. Res. 57, 557–571. doi: 10.1055/a-2720-5398, 41213606

[ref10] Balbim G. M. Boa Sorte Silva N. C. Ten Brinke L. Falck R. S. Hortobágyi T. Granacher U. . (2024). Aerobic exercise training effects on hippocampal volume in healthy older individuals: a meta-analysis of randomized controlled trials. Geroscience 46, 2755–2764. doi: 10.1007/s11357-023-00971-7, 37943486 PMC10828456

[ref11] Barad Z. Augusto J. Kelly Á M. (2023). Exercise-induced modulation of neuroinflammation in ageing. J. Physiol. 601, 2069–2083. doi: 10.1113/jp282894 36479905

[ref12] Baranowski B. J. Mohammad A. Finch M. S. Brown A. Dhaliwal R. Marko D. M. . (2023). Exercise training and BDNF injections alter amyloid precursor protein (APP) processing enzymes and improve cognition. J. Appl. Physiol. 135, 121–135. doi: 10.1152/japplphysiol.00114.2023, 37262102

[ref13] Benedict C. Joshi J. C. (2025). Repolarization of inflammatory macrophages into reparative stage targeting cannabinoid receptor2: a potential perspective to dampen lung injury/ARDS. Front. Pharmacol. 16:1623857. doi: 10.3389/fphar.2025.1623857, 41282589 PMC12634632

[ref14] Ben-Khemis M. Liu D. Pintard C. Song Z. Hurtado-Nedelec M. Marie J. C. . (2023). TNFα counteracts interleukin-10 anti-inflammatory pathway through the NOX2-Lyn-SHP-1 axis in human monocytes. Redox Biol. 67:102898. doi: 10.1016/j.redox.2023.102898, 37757542 PMC10539668

[ref15] Biazus-Sehn L. F. Schuch F. B. Firth J. de Souza Stigger F. (2020). Effects of physical exercise on cognitive function of older adults with mild cognitive impairment: a systematic review and meta-analysis. Arch. Gerontol. Geriatr. 89:104048. doi: 10.1016/j.archger.2020.104048, 32460123

[ref16] Bower J. E. Kuhlman K. R. (2023). Psychoneuroimmunology: An introduction to immune-to-brain communication and its implications for clinical psychology. Annu. Rev. Clin. Psychol. 19, 331–359. doi: 10.1146/annurev-clinpsy-080621-045153, 36791765

[ref17] Bycura D. Santos A. C. Shiffer A. Kyman S. Winfree K. Sutliffe J. . (2021). Impact of different exercise modalities on the human gut microbiome. Sports (Basel) 9:14. doi: 10.3390/sports9020014, 33494210 PMC7909775

[ref18] Cammisuli D. M. Fusi J. Scarfò G. Daniele S. Castelnuovo G. Franzoni F. (2022). A minireview exploring the interplay of the muscle-gut-brain (MGB) Axis to improve knowledge on mental disorders: implications for clinical neuroscience research and therapeutics. Oxidative Med. Cell. Longev. 2022:8806009. doi: 10.1155/2022/8806009, 36160716 PMC9499796

[ref19] Caputi V. Popov J. Giron M. C. S O. A. M. (2021). Gut microbiota as a mediator of host neuro-immune interactions: implications in neuroinflammatory disorders. Mod. Trends Psychiatry 32, 40–57. doi: 10.1159/000510416 34032644

[ref20] Chen See J. R. Amos D. Wright J. Lamendella R. Santanam N. (2022). Synergistic effects of exercise and catalase overexpression on gut microbiome. Environ. Microbiol. 24, 4220–4235. doi: 10.1111/1462-2920.15670, 34270161 PMC8761204

[ref21] Chen X. Xue J. Zou J. Zhao X. Li L. Jia R. . (2023). Resveratrol alleviated neuroinflammation induced by pseudorabies virus infection through regulating microglial M1/M2 polarization. Biomed. Pharmacother. 160:114271. doi: 10.1016/j.biopha.2023.114271, 36724642

[ref22] Cintado E. Muela P. Martín-Rodríguez L. Alcaide I. Tezanos P. Vlckova K. . (2025). Gut microbiota regulates exercise-induced hormetic modulation of cognitive function. EBioMedicine 119:105876. doi: 10.1016/j.ebiom.2025.105876, 40768832 PMC12789708

[ref23] Cohn-Schwartz E. (2020). Pathways from social activities to cognitive functioning: the role of physical activity and mental health. Innov. Aging 4:igaa015. doi: 10.1093/geroni/igaa015, 32665981 PMC7325149

[ref24] Cutuli D. Decandia D. Giacovazzo G. Coccurello R. (2023). Physical exercise as disease-modifying alternative against Alzheimer's disease: a gut-muscle-brain partnership. Int. J. Mol. Sci. 24:14686. doi: 10.3390/ijms241914686, 37834132 PMC10572207

[ref25] Dalton A. Mermier C. Zuhl M. (2019). Exercise influence on the microbiome-gut-brain axis. Gut Microbes 10, 555–568. doi: 10.1080/19490976.2018.1562268, 30704343 PMC6748614

[ref26] Dang R. Liu A. Zhou Y. Li X. Wu M. Cao K. . (2024). Astrocytic neuroligin 3 regulates social memory and synaptic plasticity through adenosine signaling in male mice. Nat. Commun. 15:8639. doi: 10.1038/s41467-024-52974-3, 39366972 PMC11452673

[ref27] Dastamooz S. Sadeghi-Bahmani D. Farahani M. H. D. Wong S. H. S. Yam J. C. S. Tham C. C. Y. . (2023). The efficacy of physical exercise interventions on mental health, cognitive function, and ADHD symptoms in children and adolescents with ADHD: an umbrella review. EClinicalMedicine 62:102137. doi: 10.1016/j.eclinm.2023.102137, 37599910 PMC10432969

[ref28] Dejanovic B. Sheng M. Hanson J. E. (2024). Targeting synapse function and loss for treatment of neurodegenerative diseases. Nat. Rev. Drug Discov. 23, 23–42. doi: 10.1038/s41573-023-00823-1, 38012296

[ref29] Deng S. Jin P. Liu S. He Y. Sherchan P. Zhang J. H. . (2023). Recruitment of regulatory T cells with rCCL17 promotes M2 microglia/macrophage polarization through TGFβ/TGFβR/Smad2/3 pathway in a mouse model of intracerebral hemorrhage. Exp. Neurol. 367:114451. doi: 10.1016/j.expneurol.2023.114451, 37257716

[ref30] Dhahbi W. Briki W. Heissel A. Schega L. Dergaa I. Guelmami N. . (2025). Physical activity to counter age-related cognitive decline: benefits of aerobic, resistance, and combined training-a narrative review. Sports Med. Open 11:56. doi: 10.1186/s40798-025-00857-2, 40381170 PMC12085549

[ref31] Dikiy S. Rudensky A. Y. (2023). Principles of regulatory T cell function. Immunity 56, 240–255. doi: 10.1016/j.immuni.2023.01.004, 36792571

[ref32] Dmytriv T. R. Storey K. B. Lushchak V. I. (2024). Intestinal barrier permeability: the influence of gut microbiota, nutrition, and exercise. Front. Physiol. 15:1380713. doi: 10.3389/fphys.2024.1380713, 39040079 PMC11260943

[ref33] Dohnalová L. Lundgren P. Carty J. R. E. Goldstein N. Wenski S. L. Nanudorn P. . (2022). A microbiome-dependent gut-brain pathway regulates motivation for exercise. Nature 612, 739–747. doi: 10.1038/s41586-022-05525-z, 36517598 PMC11162758

[ref34] Dos Santos J. R. Bortolanza M. Ferrari G. D. Lanfredi G. P. do Nascimento G. C. Azzolini A. E. C. S. . (2020). One-week high-intensity interval training increases hippocampal plasticity and mitochondrial content without changes in redox state. Antioxidants 9:445. doi: 10.3390/antiox9050445, 32455608 PMC7278594

[ref35] Duraj T. Kalamian M. Zuccoli G. Maroon J. C. D'Agostino D. P. Scheck A. C. . (2024). Clinical research framework proposal for ketogenic metabolic therapy in glioblastoma. BMC Med. 22:578. doi: 10.1186/s12916-024-03775-4, 39639257 PMC11622503

[ref36] Enette L. Vogel T. Merle S. Valard-Guiguet A.-G. Ozier-Lafontaine N. Neviere R. . (2020). Effect of 9 weeks continuous vs. interval aerobic training on plasma BDNF levels, aerobic fitness, cognitive capacity and quality of life among seniors with mild to moderate Alzheimer’s disease: a randomized controlled trial. Eur. Rev. Aging Phys. Act. 17:2. doi: 10.1186/s11556-019-0234-1, 31921371 PMC6945614

[ref37] Erny D. Dokalis N. Mezö C. Castoldi A. Mossad O. Staszewski O. . (2021). Microbiota-derived acetate enables the metabolic fitness of the brain innate immune system during health and disease. Cell Metab. 33, 2260–2276.e7. doi: 10.1016/j.cmet.2021.10.010, 34731656

[ref38] Faust T. E. Lee Y. H. O'Connor C. D. Boyle M. A. Gunner G. Durán-Laforet V. . (2025). Microglia-astrocyte crosstalk regulates synapse remodeling via Wnt signaling. Cell 188, 5212–5230.e21. doi: 10.1016/j.cell.2025.08.023, 40934914 PMC12489809

[ref39] Gong L. Liang J. Xie L. Zhang Z. Mei Z. Zhang W. (2024). Metabolic reprogramming in Gliocyte post-cerebral ischemia/reperfusion: from pathophysiology to therapeutic potential. Curr. Neuropharmacol. 22, 1672–1696. doi: 10.2174/1570159X22666240131121032, 38362904 PMC11284719

[ref40] Grasmann G. Mondal A. Leithner K. (2021). Flexibility and adaptation of cancer cells in a heterogenous metabolic microenvironment. Int. J. Mol. Sci. 22:1476. doi: 10.3390/ijms22031476, 33540663 PMC7867260

[ref41] Gray J. P. Müller V. I. Eickhoff S. B. Fox P. T. (2020). Multimodal abnormalities of brain structure and function in major depressive disorder: a meta-analysis of neuroimaging studies. Am. J. Psychiatry 177, 422–434. doi: 10.1176/appi.ajp.2019.19050560, 32098488 PMC7294300

[ref42] Greenhill C. (2020). Gut microbiome influences exercise response. Nat. Rev. Endocrinol. 16, 68–69. doi: 10.1038/s41574-019-0309-0, 31801995

[ref43] Guo S. Wang H. Yin Y. (2022). Microglia polarization from M1 to M2 in neurodegenerative diseases. Front. Aging Neurosci. 14:815347. doi: 10.3389/fnagi.2022.815347, 35250543 PMC8888930

[ref44] Hartmann F. J. Mrdjen D. McCaffrey E. Glass D. R. Greenwald N. F. Bharadwaj A. . (2021). Single-cell metabolic profiling of human cytotoxic T cells. Nat. Biotechnol. 39, 186–197. doi: 10.1038/s41587-020-0651-8, 32868913 PMC7878201

[ref45] Hays K. E. Pfaffinger J. M. Ryznar R. (2024). The interplay between gut microbiota, short-chain fatty acids, and implications for host health and disease. Gut Microbes 16:2393270. doi: 10.1080/19490976.2024.2393270, 39284033 PMC11407412

[ref46] He X. Jiao Y. Ma L. Zhang B. Zhu L. (2025). The impact of exercise interventions on cognitive frailty: a scoping review of outcomes and biological mechanisms. Front. Public Health 13:1738522. doi: 10.3389/fpubh.2025.1738522, 41602035 PMC12832520

[ref47] Herbert C. Meixner F. Wiebking C. Gilg V. (2020). Regular physical activity, short-term exercise, mental health, and well-being among university students: the results of an online and a laboratory study. Front. Psychol. 11:509. doi: 10.3389/fpsyg.2020.00509, 32528333 PMC7264390

[ref48] Hu D. Huang C. Tang L. Lei J. Wang J. Hu W. . (2025). NR4A2 attenuates early brain injury after intracerebral hemorrhage by promoting M2 microglial polarization via TLR4/TRAF6/NF-κB pathway inhibition. Cell. Mol. Life Sci. 82, 262–223. doi: 10.1007/s00018-025-05755-0, 40580316 PMC12206217

[ref49] Hu C. Xuan Y. Zhang X. Liu Y. Yang S. Yang K. (2022). Immune cell metabolism and metabolic reprogramming. Mol. Biol. Rep. 49, 9783–9795. doi: 10.1007/s11033-022-07474-2, 35696048 PMC9189272

[ref50] Huang H. Li W. Qin Z. Shen H. Li X. Wang W. (2021). Physical exercise increases peripheral brain-derived neurotrophic factors in patients with cognitive impairment: a meta-analysis. Restor. Neurol. Neurosci. 39, 159–171. doi: 10.3233/rnn-201060, 33998558

[ref51] Illes P. Rubini P. Ulrich H. Zhao Y. Tang Y. (2020). Regulation of microglial functions by purinergic mechanisms in the healthy and diseased CNS. Cells 9:1108. doi: 10.3390/cells9051108, 32365642 PMC7290360

[ref52] Ionescu-Tucker A. Butler C. W. Berchtold N. C. Matheos D. P. Wood M. A. Cotman C. W. (2021). Exercise reduces H3K9me3 and regulates brain derived neurotrophic factor and GABRA2 in an age dependent manner. Front. Aging Neurosci. 13:798297. doi: 10.3389/fnagi.2021.798297, 34970138 PMC8712855

[ref53] Jang J. Kim S. R. Lee J. E. Lee S. Son H. J. Choe W. . (2023). Molecular mechanisms of neuroprotection by ketone bodies and ketogenic diet in cerebral ischemia and neurodegenerative diseases. Int. J. Mol. Sci. 25:124. doi: 10.3390/ijms25010124, 38203294 PMC10779133

[ref54] Jensen N. J. Wodschow H. Z. Nilsson M. Rungby J. (2020). Effects of ketone bodies on brain metabolism and function in neurodegenerative diseases. Int. J. Mol. Sci. 21:8767. doi: 10.3390/ijms21228767, 33233502 PMC7699472

[ref55] Jing Z. Yinhang W. Jian C. Zhanbo Q. Xinyue W. Shuwen H. (2025). Interaction between gut microbiota and T cell immunity in colorectal cancer. Autoimmun. Rev. 24:103807. doi: 10.1016/j.autrev.2025.103807 40139455

[ref56] Jo D. Song J. (2021). Irisin acts via the PGC-1α and BDNF pathway to improve depression-like behavior. Clin. Nutr. Res. 10, 292–302. doi: 10.7762/cnr.2021.10.4.292, 34796134 PMC8575642

[ref57] Kamiya K. Tanaka S. Saito H. Yamashita M. Yonezawa R. Hamazaki N. . (2025). Effects of acute phase intensive exercise training in patients with acute decompensated heart failure. JACC Heart Fail. 13, 912–922. doi: 10.1016/j.jchf.2024.11.006, 39846909

[ref58] Kiran N. S. Yashaswini C. Chatterjee A. Prajapati B. (2025). Impact of gastrointestinal dysbiosis on tryptophan metabolism and neurological cancer progression. Med. Oncol. 42:412. doi: 10.1007/s12032-025-02972-2, 40770583

[ref59] Kong J. Xie Y. Fan R. Wang Q. Luo Y. Dong P. (2025). Exercise orchestrates systemic metabolic and neuroimmune homeostasis via the brain-muscle-liver axis to slow down aging and neurodegeneration: a narrative review. Eur. J. Med. Res. 30:475. doi: 10.1186/s40001-025-02751-9, 40506775 PMC12160435

[ref60] Kotecki K. Bradford M. S. (2022). Clorpactin: An alternative irrigation method for Total knee arthroplasty joint infection revisions. J. Knee Surg. 35, 874–883. doi: 10.1055/s-0040-1721087, 33231280

[ref61] Kraemer R. R. Kraemer B. R. (2023). The effects of peripheral hormone responses to exercise on adult hippocampal neurogenesis. Front. Endocrinol. 14:1202349. doi: 10.3389/fendo.2023.1202349, 38084331 PMC10710532

[ref62] Krolak T. Kaplan L. Navas K. Chen L. Birmingham A. Ryvkin D. . (2025). Brain endothelial gap junction coupling enables rapid vasodilation propagation during neurovascular coupling. Cell 188, 5003–5019.e22. doi: 10.1016/j.cell.2025.06.030, 40675149 PMC12337775

[ref63] Lao-Peregrin C. Xiang G. Kim J. Srivastava I. Fall A. B. Gerhard D. M. . (2024). Synaptic plasticity via receptor tyrosine kinase/G-protein-coupled receptor crosstalk. Cell Rep. 43:113595. doi: 10.1016/j.celrep.2023.113595, 38117654 PMC10844890

[ref64] Lee J. M. Sim T. H. Kim S. H. Choi Y. J. Lee J. H. Yeo S. G. . (2025). Exercise-induced FNDC5/irisin ameliorates cognitive impairment in aged mice, associated with antioxidant and neurotrophic responses. Antioxidants 14:1239. doi: 10.3390/antiox14101239, 41154548 PMC12561080

[ref65] Leger C. Quirié A. Méloux A. Fontanier E. Chaney R. Basset C. . (2024). Impact of exercise intensity on cerebral BDNF levels: role of FNDC5/irisin. Int. J. Mol. Sci. 25:1213. doi: 10.3390/ijms25021213, 38279218 PMC10816613

[ref66] Lewis L. Valvi D. Gedaly R. Marti F. (2025). Mitochondrial unfolded protein response in regulatory T cell function: a protective mechanism in immune aging. Front. Immunol. 16:1621759. doi: 10.3389/fimmu.2025.1621759, 40661960 PMC12256246

[ref67] Li J. Song J. Jia L. Wang M. Ji X. Meng R. . (2024). Exosomes in central nervous system diseases: a comprehensive review of emerging research and clinical Frontiers. Biomolecules 14:1519. doi: 10.3390/biom14121519, 39766226 PMC11673277

[ref68] Lin Z. Xuan Y. Zhang Y. Zhou Q. Qiu W. (2025). Hypothalamus and brainstem circuits in the regulation of glucose homeostasis. Am. J. Physiol. Endocrinol. Metab. 328, E588–e598. doi: 10.1152/ajpendo.00474.2024, 40047236

[ref69] Liu T. Li H. Colton J. P. Ge S. Li C. (2020). The BDNF Val66Met polymorphism, regular exercise, and cognition: a systematic review. West. J. Nurs. Res. 42, 660–673. doi: 10.1177/0193945920907308, 32075548

[ref70] Liu J. Liang Y. Meng Q. Chen J. Ma J. Zhu H. . (2024). Antagonism of β-arrestins in IL-4–driven microglia reactivity via the Samd4/mTOR/OXPHOS axis in Parkinson’s disease. Sci. Adv. 10:eadn4845. doi: 10.1126/sciadv.adn4845, 39167645 PMC11338239

[ref71] Liu B. Zhang Y. Yang Z. Liu M. Zhang C. Zhao Y. . (2021). ω-3 DPA protected neurons from neuroinflammation by balancing microglia M1/M2 polarizations through inhibiting NF-κB/MAPK p38 signaling and activating neuron-BDNF-PI3K/AKT pathways. Mar. Drugs 19:587. doi: 10.3390/md19110587, 34822458 PMC8619469

[ref72] Lu Y. Yin H. Lou L. Liu Z. Zhu H. Gu C. . (2025). M2 polarization of macrophages: manipulation of spinal cord injury repair. Neural Regen. Res. 21:10-4103. doi: 10.4103/NRR.NRR-D-24-01579 PMC13557674 41017703

[ref73] Ludyga S. Gerber M. Pühse U. Looser V. N. Kamijo K. (2020). Systematic review and meta-analysis investigating moderators of long-term effects of exercise on cognition in healthy individuals. Nat. Hum. Behav. 4, 603–612. doi: 10.1038/s41562-020-0851-8, 32231280

[ref74] Ma Y. Zhao W. Chen D. Zhou D. Gao Y. Bian Y. . (2023). Disinhibition of mesolimbic dopamine circuit by the lateral hypothalamus regulates pain sensation. J. Neurosci. 43, 4525–4540. doi: 10.1523/jneurosci.2298-22.2023, 37188517 PMC10278683

[ref75] Malange K. F. Navia-Pelaez J. M. Dias E. V. Lemes J. B. P. Choi S.-H. Dos Santos G. G. . (2022). Macrophages and glial cells: innate immune drivers of inflammatory arthritic pain perception from peripheral joints to the central nervous system. Front. Pain Res. 3:1018800. doi: 10.3389/fpain.2022.1018800, 36387416 PMC9644179

[ref76] Mao M. Xu Y. Zhang X.-Y. Yang L. An X.-b. Qu Y. . (2020). MicroRNA-195 prevents hippocampal microglial/macrophage polarization towards the M1 phenotype induced by chronic brain hypoperfusion through regulating CX3CL1/CX3CR1 signaling. J. Neuroinflammation 17:244. doi: 10.1186/s12974-020-01919-w, 32819407 PMC7439693

[ref77] Molska M. Mruczyk K. Cisek-Woźniak A. Prokopowicz W. Szydełko P. Jakuszewska Z. . (2024). The influence of intestinal microbiota on BDNF levels. Nutrients 16:2891. doi: 10.3390/nu16172891, 39275207 PMC11397622

[ref78] Morella I. Negro M. Dossena M. Brambilla R. D'Antona G. (2023). Gut-muscle-brain axis: molecular mechanisms in neurodegenerative disorders and potential therapeutic efficacy of probiotic supplementation coupled with exercise. Neuropharmacology 240:109718. doi: 10.1016/j.neuropharm.2023.109718, 37774944

[ref79] Moujalled D. Strasser A. Liddell J. R. (2021). Molecular mechanisms of cell death in neurological diseases. Cell Death Differ. 28, 2029–2044. doi: 10.1038/s41418-021-00814-y, 34099897 PMC8257776

[ref80] Mu Y. Yang X. Feng Y. Zhang L. Xu J. Li M. . (2025). Physical exercise promotes white matter repair after ischemic stroke. Neural Regen. Res. 21, 2397–2406. doi: 10.4103/nrr.Nrr-d-24-00861, 40313089 PMC13211827

[ref81] Murphy R. M. Watt M. J. Febbraio M. A. (2020). Metabolic communication during exercise. Nat. Metab. 2, 805–816. doi: 10.1038/s42255-020-0258-x, 32747791

[ref82] Murugathasan M. Jafari A. Amandeep A. Hassan S. A. Chihata M. Abdul-Sater A. A. (2023). Moderate exercise induces trained immunity in macrophages. Am. J. Physiol. Cell Physiol. 325, C429–c442. doi: 10.1152/ajpcell.00130.2023, 37306389

[ref83] Nicastri C. M. McFeeley B. M. Simon S. S. Ledreux A. Håkansson K. Granholm A. C. . (2022). BDNF mediates improvement in cognitive performance after computerized cognitive training in healthy older adults. Alzheimers Dement. (N Y) 8:e12337. doi: 10.1002/trc2.12337, 36089933 PMC9428279

[ref84] Okamoto T. Morino K. Ugi S. Nakagawa F. Lemecha M. Ida S. . (2019). Microbiome potentiates endurance exercise through intestinal acetate production. Am. J. Physiol. Endocrinol. Metab. 316, E956–e966. doi: 10.1152/ajpendo.00510.2018, 30860879

[ref85] Opialla T. Gollasch B. Kuich P. Klug L. Rahn G. Busjahn A. . (2022). Exercise blood-drop metabolic profiling links metabolism with perceived exertion. Front. Mol. Biosci. 9:1042231. doi: 10.3389/fmolb.2022.1042231, 36619172 PMC9822726

[ref86] Oprea S. Pantu C. Costea D. Dumitru A. V. Tataru C. I. Dobrin N. . (2025). Neurovascular Signaling at the gliovascular Interface: from flow regulation to cognitive energy coupling. Int. J. Mol. Sci. 27:69. doi: 10.3390/ijms27010069, 41515949 PMC12785549

[ref87] Oyovwi M. O. Ogenma U. T. Onyenweny A. (2025). Exploring the impact of exercise-induced BDNF on neuroplasticity in neurodegenerative and neuropsychiatric conditions. Mol. Biol. Rep. 52:140. doi: 10.1007/s11033-025-10248-1 39832087

[ref88] Pang Q.-M. Chen S.-Y. Fu S.-P. Zhou H. Zhang Q. Ao J. . (2022). Regulatory role of mesenchymal stem cells on secondary inflammation in spinal cord injury. J. Inflamm. Res. 15, 573–593. doi: 10.2147/JIR.S349572, 35115806 PMC8802142

[ref89] Papp G. Szabó K. Jámbor I. Mile M. Berki A. R. Arany A. C. . (2021). Regular exercise may restore certain age-related alterations of adaptive immunity and rebalance immune regulation. Front. Immunol. 12:639308. doi: 10.3389/fimmu.2021.639308, 33936054 PMC8085426

[ref90] Pasquerella L. Aychman M. M. Tasnim N. Piccirillo A. Holmes V. Basso J. C. (2025). The benefits of chronic sport participation and acute exercise on mental health and executive functioning in adolescents. Sci. Rep. 15:22392. doi: 10.1038/s41598-025-88427-0, 40594821 PMC12216928

[ref91] Pignataro P. Dicarlo M. Zerlotin R. Zecca C. Dell’Abate M. T. Buccoliero C. . (2021). FNDC5/irisin system in neuroinflammation and neurodegenerative diseases: update and novel perspective. Int. J. Mol. Sci. 22:1605. doi: 10.3390/ijms22041605, 33562601 PMC7915567

[ref92] Plourde G. Roumes H. Suissa L. Hirt L. Doche É. Pellerin L. . (2024). Neuroprotective effects of lactate and ketone bodies in acute brain injury. J. Cereb. Blood Flow Metab. 44, 1078–1088. doi: 10.1177/0271678X241245486, 38603600 PMC11179615

[ref93] Portincasa P. Bonfrate L. Vacca M. De Angelis M. Farella I. Lanza E. . (2022). Gut microbiota and Short chain fatty acids: implications in glucose homeostasis. Int. J. Mol. Sci. 23:1105. doi: 10.3390/ijms23031105, 35163038 PMC8835596

[ref94] Pujari V. (2024). Moving to improve mental health - the role of exercise in cognitive function: a narrative review. J. Pharm. Bioallied Sci. 16, S26–s30. doi: 10.4103/jpbs.jpbs_614_23, 38595617 PMC11000952

[ref95] Qiao Y. Cheng R. Li X. Zheng H. Guo J. Wei L. . (2025). Plateau environment, gut microbiota, and depression: a possible concealed connection? Curr. Issues Mol. Biol. 47:487. doi: 10.3390/cimb47070487, 40728956 PMC12293327

[ref96] Qu S. Hu S. Xu H. Wu Y. Ming S. Zhan X. . (2024). TREM-2 drives development of multiple sclerosis by promoting pathogenic Th17 polarization. Neurosci. Bull. 40, 17–34. doi: 10.1007/s12264-023-01094-x, 37498431 PMC10774236

[ref97] Quaresma M. Mancin L. Paoli A. Mota J. F. (2024). The interplay between gut microbiome and physical exercise in athletes. Curr. Opin. Clin. Nutr. Metab. Care 27, 428–433. doi: 10.1097/mco.0000000000001056, 39083429

[ref98] Radin D. P. Tsirka S. E. (2020). Interactions between tumor cells, neurons, and microglia in the glioma microenvironment. Int. J. Mol. Sci. 21:8476. doi: 10.3390/ijms21228476, 33187183 PMC7698134

[ref99] Raut S. Cucullo L. (2025). Antidiabetic agents as antioxidant and anti-inflammatory therapies in neurological and cardiovascular diseases. Antioxidants 14:1490. doi: 10.3390/antiox14121490, 41462689 PMC12729538

[ref100] Ren C. Li D. Zhou Q. Hu X. (2020). Mitochondria-targeted TPP-MoS2 with dual enzyme activity provides efficient neuroprotection through M1/M2 microglial polarization in an Alzheimer's disease model. Biomaterials 232:119752. doi: 10.1016/j.biomaterials.2019.119752 31923845

[ref101] Ribeiro F. M. Silva M. A. Lyssa V. Marques G. Lima H. K. Franco O. L. . (2022). The molecular signaling of exercise and obesity in the microbiota-gut-brain axis. Front. Endocrinol. (Lausanne) 13:927170. doi: 10.3389/fendo.2022.927170, 35966101 PMC9365995

[ref102] Rocamora-Reverte L. Villunger A. Wiegers G. J. (2022). Cell-specific immune regulation by glucocorticoids in murine models of infection and inflammation. Cells 11:2126. doi: 10.3390/cells11142126, 35883569 PMC9324070

[ref103] Rogovskii V. (2020). Immune tolerance as the physiologic counterpart of chronic inflammation. Front. Immunol. 11:2061. doi: 10.3389/fimmu.2020.02061, 33117330 PMC7561427

[ref104] Ronaldson P. T. Davis T. P. (2020). Regulation of blood–brain barrier integrity by microglia in health and disease: a therapeutic opportunity. J. Cereb. Blood Flow Metab. 40, S6–S24. doi: 10.1177/0271678X20951995, 32928017 PMC7687032

[ref105] Santos E. A. Silva J. L. Leocádio P. C. L. Andrade M. E. R. Queiroz-Junior C. M. Oliveira N. S. S. . (2024). Cutaneous application of capsaicin cream reduces clinical signs of experimental colitis and repairs intestinal barrier integrity by modulating the gut microbiota and tight junction proteins. ACS Pharmacol. Transl. Sci. 7, 2143–2153. doi: 10.1021/acsptsci.4c00207, 39022369 PMC11249629

[ref106] Saponaro F. Bertolini A. Baragatti R. Galfo L. Chiellini G. Saba A. . (2024). Myokines and microbiota: new perspectives in the endocrine muscle-gut axis. Nutrients 16:4032. doi: 10.3390/nu16234032, 39683426 PMC11643575

[ref107] Saxton R. A. Tsutsumi N. Su L. L. Abhiraman G. C. Mohan K. Henneberg L. T. . (2021). Structure-based decoupling of the pro- and anti-inflammatory functions of interleukin-10. Science 371:eabc8433. doi: 10.1126/science.abc8433, 33737461 PMC9132103

[ref108] Schlegel P. Novotny M. Klimova B. Valis M. (2019). "muscle-gut-brain Axis": can physical activity help patients with Alzheimer's disease due to microbiome modulation? J Alzheimer's Dis 71, 861–878. doi: 10.3233/jad-190460, 31476155

[ref109] Scriven M. McSweeney A. O'Carroll T. Morkl S. Butler M. I. (2023). The muscle-gut-brain axis and psychiatric illness. Adv. Biol. 7:e2200214. doi: 10.1002/adbi.202200214, 37080945

[ref110] Segui-Perez C. Stapels D. A. C. Ma Z. Su J. Passchier E. Westendorp B. . (2024). MUC13 negatively regulates tight junction proteins and intestinal epithelial barrier integrity via protein kinase C. J. Cell Sci. 137:jcs261468. doi: 10.1242/jcs.261468, 38345099 PMC10984281

[ref111] Seo D. Y. Heo J. W. Ko J. R. Kwak H. B. (2019). Exercise and neuroinflammation in health and disease. Int. Neurourol. J. 23, S82–S92. doi: 10.5213/inj.1938214.107, 31795607 PMC6905205

[ref112] Shi J. Hu Z. Y. Wen Y. R. Wang Y. F. Lin Y. Y. Zhao H. Z. . (2022). Optimal modes of mind-body exercise for treating chronic non-specific low back pain: systematic review and network meta-analysis. Front. Neurosci. 16:1046518. doi: 10.3389/fnins.2022.1046518, 36466167 PMC9713308

[ref113] Shin H. E. Kwak S. E. Lee J. H. Zhang D. Bae J. H. Song W. (2019). Exercise, the gut microbiome, and frailty. Ann. Geriatr. Med. Res. 23, 105–114. doi: 10.4235/agmr.19.0014, 32743298 PMC7370771

[ref114] Short A. K. Bui V. Zbukvic I. C. Hannan A. J. Pang T. Y. Kim J. H. (2022). Sex-dependent effects of chronic exercise on cognitive flexibility but not hippocampal Bdnf in aging mice. Neuronal Signal. 6:Ns20210053. doi: 10.1042/ns20210053, 35036000 PMC8734434

[ref115] Smith P. J. Merwin R. M. (2021). The role of exercise in management of mental health disorders: an integrative review. Annu. Rev. Med. 72, 45–62. doi: 10.1146/annurev-med-060619-022943, 33256493 PMC8020774

[ref116] Song M.-K. Jo H.-S. Kim E.-J. Kim J.-K. Lee S.-G. (2024). Gene expression of neurogenesis related to exercise intensity in a cerebral infarction rat model. Int. J. Mol. Sci. 25:8997. doi: 10.3390/ijms25168997, 39201683 PMC11354542

[ref117] Souza P. B. de Araujo Borba L. de Castro Jesus L. Valverde A. P. Gil-Mohapel J. Rodrigues A. L. S. (2023). Major depressive disorder and gut microbiota: role of physical exercise. Int. J. Mol. Sci. 24:16870. doi: 10.3390/ijms242316870 38069198 PMC10706777

[ref118] Spanaki C. Sidiropoulou K. Petraki Z. Diskos K. Konstantoudaki X. Volitaki E. . (2024). Glutamate-specific gene linked to human brain evolution enhances synaptic plasticity and cognitive processes. Iscience 27:108821. doi: 10.1016/j.isci.2024.108821, 38333701 PMC10850756

[ref119] Stojanovic B. Milivojcevic Bevc I. Dimitrijevic Stojanovic M. Stojanovic B. S. Lazarevic T. Spasic M. . (2025). Oxidative stress, inflammation, and cellular senescence in neuropathic pain: mechanistic crosstalk. Antioxidants 14:1166. doi: 10.3390/antiox14101166, 41154475 PMC12561125

[ref120] Su Y. Su Z. (2025). Effects of exercise on neuroinflammation in age-related neurodegenerative disorders. Eur. J. Med. Res. 30:909. doi: 10.1186/s40001-025-03165-3, 41024269 PMC12482578

[ref121] Sun W. Bai Z. Zhou F. (2023). The microbiota-gut-brain axis regulates motivation for exercise. MedComm 4:e304. doi: 10.1002/mco2.304, 37323875 PMC10264927

[ref122] Thaiss C. A. (2023). A microbiome exercise. Science 381:38. doi: 10.1126/science.adi6329 37410844

[ref123] Tian Z. Ji X. Liu J. (2022). Neuroinflammation in vascular cognitive impairment and dementia: current evidence, advances, and prospects. Int. J. Mol. Sci. 23:6224. doi: 10.3390/ijms23116224, 35682903 PMC9181710

[ref124] Tsilingiris D. Tzeravini E. Koliaki C. Dalamaga M. Kokkinos A. (2021). The role of mitochondrial adaptation and metabolic flexibility in the pathophysiology of obesity and insulin resistance: an updated overview. Curr. Obes. Rep. 10, 191–213. doi: 10.1007/s13679-021-00434-0, 33840072

[ref125] Tuan S. H. Chang L. H. Sun S. F. Li C. H. Chen G. B. Tsai Y. J. (2024). Assessing the clinical effectiveness of an exergame-based exercise training program using ring fit adventure to prevent and postpone frailty and sarcopenia among older adults in rural Long-term care facilities: randomized controlled trial. J. Med. Internet Res. 26:e59468. doi: 10.2196/59468, 39024000 PMC11294767

[ref126] Tucci S. Alatibi K. I. Wehbe Z. (2021). Altered metabolic flexibility in inherited metabolic diseases of mitochondrial fatty acid metabolism. Int. J. Mol. Sci. 22:3799. doi: 10.3390/ijms22073799, 33917608 PMC8038842

[ref127] Valk S. L. Xu T. Paquola C. Park B.-y. Bethlehem R. A. de Vos Wael R. . (2022). Genetic and phylogenetic uncoupling of structure and function in human transmodal cortex. Nat. Commun. 13:2341. doi: 10.1038/s41467-022-29886-1, 35534454 PMC9085871

[ref128] Verbrugghe J. Agten A. Stevens S. Hansen D. Demoulin C. Eijnde B. O. . (2020). High intensity training to treat chronic nonspecific low Back pain: effectiveness of various exercise modes. J. Clin. Med. 9:2401. doi: 10.3390/jcm9082401, 32727108 PMC7465397

[ref129] Versele R. Corsi M. Fuso A. Sevin E. Businaro R. Gosselet F. . (2020). Ketone bodies promote amyloid-β1–40 clearance in a human in vitro blood–brain barrier model. Int. J. Mol. Sci. 21:934. doi: 10.3390/ijms21030934, 32023814 PMC7037612

[ref130] Vezzoli E. Cali C. De Roo M. Ponzoni L. Sogne E. Gagnon N. . (2020). Ultrastructural evidence for a role of astrocytes and glycogen-derived lactate in learning-dependent synaptic stabilization. Cereb. Cortex 30, 2114–2127. doi: 10.1093/cercor/bhz226, 31807747 PMC7174989

[ref131] Waddington E. E. Allison D. J. Calabrese E. M. Pekos C. Lee A. Walsh J. J. . (2024). Orienteering combines vigorous-intensity exercise with navigation to improve human cognition and increase brain-derived neurotrophic factor. PLoS One 19:e0303785. doi: 10.1371/journal.pone.0303785, 38776348 PMC11111042

[ref132] Wan Y. Cao C. Zeng W. (2025). The sympathetic neurons in the gut: perspectives on metabolic and immune health and diseases. Curr. Opin. Neurobiol. 93:103051. doi: 10.1016/j.conb.2025.103051, 40446451

[ref133] Wang Y. Chen J. Ni Y. Liu Y. Gao X. Tse M. A. . (2024). Exercise-changed gut mycobiome as a potential contributor to metabolic benefits in diabetes prevention: an integrative multi-omics study. Gut Microbes 16:2416928. doi: 10.1080/19490976.2024.2416928, 39473051 PMC11533799

[ref134] Wang Z. Emmerich A. Pillon N. J. Moore T. Hemerich D. Cornelis M. C. . (2022). Genome-wide association analyses of physical activity and sedentary behavior provide insights into underlying mechanisms and roles in disease prevention. Nat. Genet. 54, 1332–1344. doi: 10.1038/s41588-022-01165-1, 36071172 PMC9470530

[ref135] Wang Q. Yao H. Liu W. Ya B. Cheng H. Xing Z. . (2021). Microglia polarization in Alzheimer’s disease: mechanisms and a potential therapeutic target. Front. Aging Neurosci. 13:772717. doi: 10.3389/fnagi.2021.772717, 34819850 PMC8606412

[ref136] Watson N. McClelland T. J. Puthucheary Z. (2023). Is there a role for ketones as alternative fuel in critical illness? Curr. Opin. Crit. Care 29, 300–305. doi: 10.1097/mcc.0000000000001061, 37306537

[ref137] Wen X. Fan J. Duan X. Zhu X. Bai J. Zhang T. (2025). Mitochondrial DNA in exercise-mediated innate immune responses. Int. J. Mol. Sci. 26:3069. doi: 10.3390/ijms26073069, 40243714 PMC11988935

[ref138] Xia H. H. Huang J. Z. (2025). Tryptophan metabolism at the crossroads of the neuro-immuno-microbial axis: implications for precision medicine in chronic diseases. Front. Cell. Infect. Microbiol. 15:1707850. doi: 10.3389/fcimb.2025.1707850, 41602107 PMC12833013

[ref139] Xu Q. Fan Y. Zhu J. Wang X. (2024). The effect of different exercise on physical fitness, cognition, and mental health in healthy older adults. Heliyon 10:e36510. doi: 10.1016/j.heliyon.2024.e36510, 39253255 PMC11382082

[ref140] Yang P. Chen H. Wang T. Su H. Li J. He Y. . (2023). Electroacupuncture promotes synaptic plasticity in rats with chronic inflammatory pain-related depression by upregulating BDNF/TrkB/CREB signaling pathway. Brain Behav. 13:e3310. doi: 10.1002/brb3.3310, 37948105 PMC10726860

[ref141] Yang W. Liu H. Xu L. Yu T. Zhao X. Yao S. . (2022). GPR120 inhibits colitis through regulation of CD4(+) T cell interleukin 10 production. Gastroenterology 162, 150–165. doi: 10.1053/j.gastro.2021.09.018, 34536451 PMC8678294

[ref142] Yang W. Liu Y. Yang G. Meng B. Yi Z. Yang G. . (2021). Moderate-intensity physical exercise affects the exercise performance and gut microbiota of mice. Front. Cell. Infect. Microbiol. 11:712381. doi: 10.3389/fcimb.2021.712381, 34631598 PMC8498591

[ref143] Yao Z. F. Hsieh S. Yang M. H. (2024). Exercise habits and mental health: exploring the significance of multimodal imaging markers. Prog. Brain Res. 286, 179–209. doi: 10.1016/bs.pbr.2023.11.001, 38876575

[ref144] Yin Y. Guo Q. Zhou X. Duan Y. Yang Y. Gong S. . (2022). Role of brain-gut-muscle axis in human health and energy homeostasis. Front. Nutr. 9:947033. doi: 10.3389/fnut.2022.947033, 36276808 PMC9582522

[ref145] Yin F. He Y. Li J. Gao Y. (2025). Immune cell senescence in autoimmunity: implications for disease pathogenesis and therapeutic targeting. Front. Immunol. 16:1596686. doi: 10.3389/fimmu.2025.1596686, 40852730 PMC12367673

[ref146] Ying W. Weng W. Wang P. Pan C. Qiu J. Huang Q. . (2025). N-Lactoyl-phenylalanine modulates lipid metabolism in microglia/macrophage via the AMPK-PGC1α-PPARγ pathway to promote recovery in mice with spinal cord injury. J. Neuroinflammation 22:167. doi: 10.1186/s12974-025-03495-3, 40579710 PMC12205500

[ref147] Yu Y. Chen K. (2025). Peripheral immune and metabolic regulation of aβ and tau by exercise in Alzheimer's disease. Front. Immunol. 16:1678526. doi: 10.3389/fimmu.2025.1678526, 41169370 PMC12568357

[ref148] Yu C. J. Wang M. Li R. Y. Wei T. Yang H. C. Yin Y. S. . (2023). TREM2 and microglia contribute to the synaptic plasticity: from physiology to pathology. Mol. Neurobiol. 60, 512–523. doi: 10.1007/s12035-022-03100-1, 36318443

[ref149] Yu Y. B. Zhao D. Y. Qi Q. Q. Long X. Li X. Chen F. X. . (2017). BDNF modulates intestinal barrier integrity through regulating the expression of tight junction proteins. Neurogastroenterol. Motil. 29:e12967. doi: 10.1111/nmo.12967, 27747999

[ref150] Zalouli V. Rajavand H. Bayat M. Khaleghnia J. Sharifianjazi F. Jafarinazhad F. . (2023). Adult hippocampal neurogenesis (AHN) controls central nervous system and promotes peripheral nervous system regeneration via physical exercise. Biomed. Pharmacother. 165:115078. doi: 10.1016/j.biopha.2023.115078, 37390707

[ref151] Zare N. Bishop D. J. Levinger I. Febbraio M. A. Broatch J. R. (2025). Exercise intensity matters: a review on evaluating the effects of aerobic exercise intensity on muscle-derived neuroprotective myokines. Alzheimers Dement. (N Y) 11:e70056. doi: 10.1002/trc2.70056, 39975467 PMC11837734

[ref152] Zhang Z. Jiang J. He Y. Cai J. Xie J. Wu M. . (2022). Pregabalin mitigates microglial activation and neuronal injury by inhibiting HMGB1 signaling pathway in radiation-induced brain injury. J. Neuroinflammation 19:231. doi: 10.1186/s12974-022-02596-7, 36131309 PMC9490947

[ref153] Zhang L. Lu X. Gong L. Cui L. Zhang H. Zhao W. . (2021). Tetramethylpyrazine protects blood-spinal cord barrier integrity by modulating microglia polarization through activation of STAT3/SOCS3 and inhibition of NF-к B signaling pathways in experimental autoimmune encephalomyelitis mice. Cell. Mol. Neurobiol. 41, 717–731. doi: 10.1007/s10571-020-00878-3, PMC11448626 32424774

[ref154] Zhang S. Zhang L. Guan N. Feng X. Lu M. Wang Y. (2025). Unveiling the role of TAM-derived extracellular vesicles in glioma progression through Treg polarization and immune suppression. Oncogene 44, 3364–3385. doi: 10.1038/s41388-025-03497-8, 40681801

[ref155] Zhao J. L. Jiang W. T. Wang X. Cai Z. D. Liu Z. H. Liu G. R. (2020). Exercise, brain plasticity, and depression. CNS Neurosci. Ther. 26, 885–895. doi: 10.1111/cns.13385, 32491278 PMC7415205

[ref156] Zhao Q. K. Ning Y. X. Xu T. C. Zhao Z. A. Pei Y. F. Niu J. Y. . (2025). Electroacupuncture alleviate brain injury through vagus nerve activation and gut microbiota in a rat model of ischemic stroke. J. Am. Heart Assoc. 15:e045929. doi: 10.1161/JAHA.125.045929 PMC12909005 41211649

[ref157] Zheng Z. V. Lyu H. Lam S. Y. E. Lam P. K. Poon W. S. Wong G. K. C. (2020). The dynamics of microglial polarization reveal the resident neuroinflammatory responses after subarachnoid hemorrhage. Transl. Stroke Res. 11, 433–449. doi: 10.1007/s12975-019-00728-5, 31628642

[ref158] Zhou R. Chen J. Tang Y. Wei C. Yu P. Ding X. . (2025). Multi-omics uncovers immune-modulatory molecules in plasma contributing to resistance exercise-ameliorated locomotor disability after incomplete spinal cord injury. Genome Med. 17:10. doi: 10.1186/s13073-025-01434-8, 39910614 PMC11796186

[ref159] Zhou Q. Deng J. Pan X. Meng D. Zhu Y. Bai Y. . (2022). Gut microbiome mediates the protective effects of exercise after myocardial infarction. Microbiome 10:82. doi: 10.1186/s40168-022-01271-6, 35637497 PMC9153113

